# Species richness stabilizes productivity via asynchrony and drought-tolerance diversity in a large-scale tree biodiversity experiment

**DOI:** 10.1126/sciadv.abk1643

**Published:** 2021-12-17

**Authors:** Florian Schnabel, Xiaojuan Liu, Matthias Kunz, Kathryn E. Barry, Franca J. Bongers, Helge Bruelheide, Andreas Fichtner, Werner Härdtle, Shan Li, Claas-Thido Pfaff, Bernhard Schmid, Julia A. Schwarz, Zhiyao Tang, Bo Yang, Jürgen Bauhus, Goddert von Oheimb, Keping Ma, Christian Wirth

**Affiliations:** 1 German Centre for Integrative Biodiversity Research (iDiv) Halle-Jena-Leipzig, Leipzig, Germany.; 2 Systematic Botany and Functional Biodiversity, Leipzig University, Leipzig, Germany.; 3 State Key Laboratory of Vegetation and Environmental Change, Institute of Botany, Chinese Academy of Sciences, Beijing, China.; 4 Institute of General Ecology and Environmental Protection, Technische Universität Dresden, Tharandt, Germany.; 5 Ecology and Biodiversity, Institute of Environmental Biology, Department of Biology, Utrecht University, Utrecht, Netherlands.; 6 Institute of Biology/Geobotany and Botanical Garden, Martin Luther University Halle-Wittenberg, Halle (Saale), Germany.; 7 Institute of Ecology, Leuphana University of Lüneburg, Lüneburg, Germany.; 8 Department of Geography, Remote Sensing Laboratories, University of Zurich, Zurich, Switzerland.; 9 Chair of Silviculture, Institute of Forest Sciences, Freiburg University, Freiburg, Germany.; 10 Institute of Ecology, College of Urban and Environmental Sciences, and Key Laboratory for Earth Surface Processes of the Ministry of Education, Peking University, Beijing, China.; 11 Institute of Biology, Geobotany and Botanical Garden, Jingdezhen University, Jiangxi, China.; 12 Max Planck Institute for Biogeochemistry, Jena, Germany.

## Abstract

Extreme climatic events threaten forests and their climate mitigation potential globally. Understanding the drivers promoting ecosystem stability is therefore considered crucial for mitigating adverse climate change effects on forests. Here, we use structural equation models to explain how tree species richness, asynchronous species dynamics, species-level population stability, and drought-tolerance traits relate to the stability of forest productivity along an experimentally manipulated species richness gradient ranging from 1 to 24 tree species. Tree species richness improved community stability by increasing asynchrony. That is, at higher species richness, interannual variation in productivity among tree species buffered the community against stress-related productivity declines. This effect was positively related to variation in stomatal control and resistance-acquisition strategies among species, but not to the community-weighted means of these trait syndromes. The identified mechanisms by which tree species richness stabilizes forest productivity emphasize the importance of diverse, mixed-species forests to adapt to climate change.

## Teaser

Species richness promotes forest stability via enhanced asynchrony, which is positively related to drought-tolerance diversity.

## INTRODUCTION

Climate change is increasing the frequency and severity of droughts and other extreme events, threatening tree growth and survival globally (*1*), including in humid tropical and subtropical forests (*2*). This compromises the ability of the world’s forests to act as carbon sinks (*3*) and as nature-based solutions to climate change mitigation (*4*). Stability, the ability of forests to maintain functioning over time and in the face of environmental stressors, is consequently emerging as a primary focus of forest management in the 21st century. One key management strategy to enhance stability may be to increase tree species richness in secondary and plantation forests (*5*, *6*).

There is compelling evidence that species richness can stabilize community biomass production against variable climate conditions such as droughts or extremely wet years (*7*–*10*). However, most of this evidence comes from grassland ecosystems. Biodiversity-stability relationships likely differ between forests and grasslands because trees invest in long-lasting structures and community composition changes more slowly in forests (*5*). The few existing studies in forests support the notion that species richness stabilizes community aboveground wood production, hereafter referred to as “productivity,” of mixed-species tree communities (*5*, *11*–*13*). However, we lack a comprehensive understanding of the underlying mechanisms that drive these biodiversity-stability relationships in forest ecosystems.

According to the insurance hypothesis (*14*), a mixture of tree species with different strategies should help to maintain or increase the functioning of forests under highly variable climatic conditions, thus increasing their temporal stability. This stability (*15*) is often quantified as temporal mean productivity (μ) divided by the temporal SD in community productivity (σ) [e.g., (*7*, *8*)] and may be promoted in mixed-species tree communities via species richness increasing performance (increasing μ) or buffering variation (decreasing σ) (*14*). Increased performance (i.e., higher productivity) at higher species richness—often called “overyielding”—has been reported by numerous studies in natural and experimental forests (*6*, *16*, *17*). Here, different species perform relatively better in mixtures than in monocultures, for example, through complementary resource use or facilitation and this higher performance can increase community stability (*5*). Second, decreased temporal variation in productivity through buffering of the effects of environmental stress may increase community stability. In contrast to overyielding, little is known about this buffering effect of biodiversity in forest ecosystems. Various mechanisms may decrease temporal variation in productivity at higher species richness (*14*, *15*, *18*, *19*), but arguably, the one most supported by theoretical and observational studies in grasslands and increasingly also in forests is species asynchrony (*7*, *18*, *20*). In forests, these asynchronous interannual dynamics in species productivity (hereafter “asynchrony”) (*19*) have been found to be the key driver of diversity effects on community stability (*5*, *11*–*13*, *21*).

**Fig. 1.**
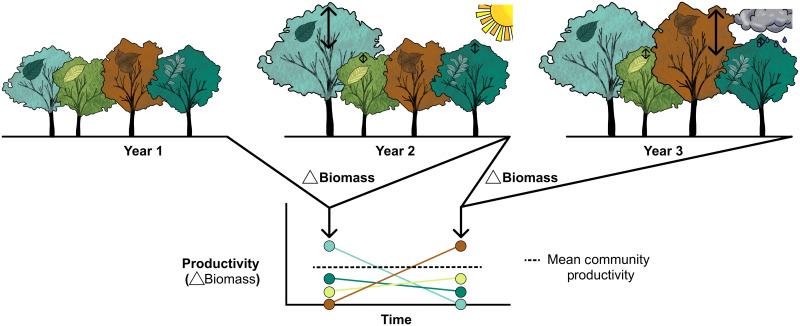
Graphical illustration of asynchronous species responses in mixed-species tree communities to contrasting climatic conditions. The tree community experiences a “normal” (year 1), an exceptionally dry (year 2), and an exceptionally wet (year 3) year, which result in distinctly different productivity responses of the participating species but the same community productivity due to compensatory dynamics. In our hypothetical example taken from a four-species mixture in the BEF-China experiment, one species (*Nyssa sinensis*, light turquoise) does not close its stomata fast during water shortage (water spender) and might continue to grow well during drought, a second species (*Liquidambar formosana*, brown) exhibits a fast down-regulation of stomatal conductance at increasing water pressure deficits and its productivity is thus more strongly reduced during drought (water saver), while the two other species (*Castanea henryi* and *Sapindus saponaria*) do not show strong reactions to the changing climatic conditions. The reverse response pattern is found during an exceptionally wet year. We hypothesize here that such asynchronous species dynamics are the key driver behind stabilizing effects of species richness on productivity in mixed-species forests and that the functional traits of coexisting species—especially those associated with drought tolerance—may help to elucidate the mechanisms that produce this asynchrony.

Asynchronous species dynamics may result from intrinsic rhythms such as phenology or mast seeding (*22*, *23*), differential responses of species to extrinsic factors such as climatic conditions (*19*, *24*), demographic stochasticity (*25*), and species interactions in mixtures like resource partitioning or biotic feedbacks (*26*, *27*). Asynchrony may buffer the temporal variation in community productivity during times of stress as some species likely maintain functioning or compensate for the productivity losses of other species (Fig. 1). Next to asynchrony, according to recent theory, the second key driver of community stability is the average stability at the population level (*28*). However, whether this average species-level population stability (hereafter “population stability”) mediates effects of species richness on community stability and whether it affects the mean or the variation in productivity is less clear. A recent meta-analysis suggests a preponderance of destabilizing effects of species richness on population stability in terrestrial ecosystems, but there are also many studies reporting stabilizing effects (*29*). For forests, positive (*5*), neutral (*11*), and negative (*12*) effects of species richness on population stability have been reported. Hence, if species richness were to stabilize community productivity via asynchrony and population stability, this could result from positive effects of asynchrony counteracting negative effects of population stability, a positive effect of either while the other has no effect, or a joint positive effect of both (*28*). Understanding these potentially stabilizing effects is especially important in the context of the global increase in the severity and frequency of extreme climatic events such as drought (*30*, *31*). Hence, there is an urgent need to identify the characteristics that allow tree species and species mixtures to maintain functioning under future global change.

While the number of species may increase community stability, communities also require certain functional characteristics to respond to variable climatic conditions such as drought stress. Two key strategies that determine a tree’s response to drought are stomatal control and cavitation resistance (*24*). First, tree species may exhibit different strategies of stomatal control. Some rely on continued water extraction and keep their stomata open, i.e., they continue to transpire although this poses a high risk for cavitation-induced mortality under extreme drought (called water spenders or anisohydric species) (*24*, *32*). Other tree species quickly decrease their stomatal conductance during water shortage to avoid transpiration losses and xylem cavitation but may risk carbon starvation under prolonged droughts (called water savers or isohydric species). Consistent with recent perspectives (*32*), we view stomatal control here along a gradient from water-spending to water-saving species behavior and quantify it through physiological traits such as stomatal conductance and control of conductance under increasing water pressure deficits (*33*, *34*). Second, drought tolerance depends on xylem resistance to cavitation because embolism decreases water availability and may ultimately lead to desiccation and tree death (*2*, *24*). Here, we use the threshold at which 50% of xylem conductivity is lost because of cavitation (Ψ_50_; measured as water potential) as a key trait (*2*) to quantify drought resistance. In addition, considering classic traits of the leaf economics spectrum (indicating conservative versus acquisitive resource use) (*35*), which were shown to correlate with cavitation resistance (*33*, *36*), may help us to understand which trait syndromes govern forest responses to variable climatic conditions such as drought stress and how drought tolerance is linked to broader dimensions of ecological variation (*32*, *37*). Hereafter, we refer to stomatal control and resistance-acquisition traits, which both are related to drought tolerance, collectively as “drought-tolerance” traits.

These drought-tolerance strategies may enable mixed-species forests to stabilize community productivity in two ways. First, tree species richness may increase community stability indirectly via promoting asynchrony through functional diversity in traits related to drought tolerance (hereafter “drought-tolerance diversity”). The importance of tree species richness and asynchrony for community stability is supported by previous studies (*5*, *11*–*13*). However, these studies were based on observational data from naturally assembled forests [with only one exception (*13*)], and tree species richness gradients were low. Therefore, it remains difficult to establish causal relationships between tree diversity and community stability. In particular, the mechanistic links between tree species richness, asynchrony and community stability, as well as the underlying trait-based mechanisms remain unknown for forests. Second, community stability could also be influenced by the community-weighted means (CWMs) of drought-tolerance traits, as indicated by findings in grassland diversity experiments where community stability was higher in communities dominated by species with traits associated with conservative resource use (*7*). It is conceivable that this effect of CWM traits should influence community stability via effects on average population stability as population stability can be influenced by species’ traits (*38*).

We use structural equation models (SEMs) to test the direct and indirect effects of species richness, asynchrony, population stability, drought-tolerance diversity, and the CWMs of drought-tolerance traits on the stability of community productivity over 10 years under the controlled conditions of a large-scale tree biodiversity experiment [BEF-China experiment (Biodiversity–Ecosystem Functioning Experiment China)] (*16*, *39*). Our experiment is located in the highly diverse subtropical forests of China and features a gradient of species richness ranging from monocultures up to mixtures of 24 tree species planted at two sites using multiple species pools. All species occurred at all richness levels, thus avoiding any confounding effects between species occurrence and richness. In our study, stomatal control and resistance-acquisition traits form two orthogonal dimensions in drought-tolerance strategies (fig. S1), which allows us to quantify the relative contributions of these trait gradients to community stability, asynchrony, and population stability. Specifically, we tested the following hypotheses:

H1: Tree species richness increases community stability via asynchrony and population stability.

H2: Diversity in stomatal control and resistance-acquisition strategies is positively related to community stability via asynchrony.

H3: CWMs of stomatal control and resistance-acquisition strategies are related to community stability via population stability.

## RESULTS

**Fig. 2.**
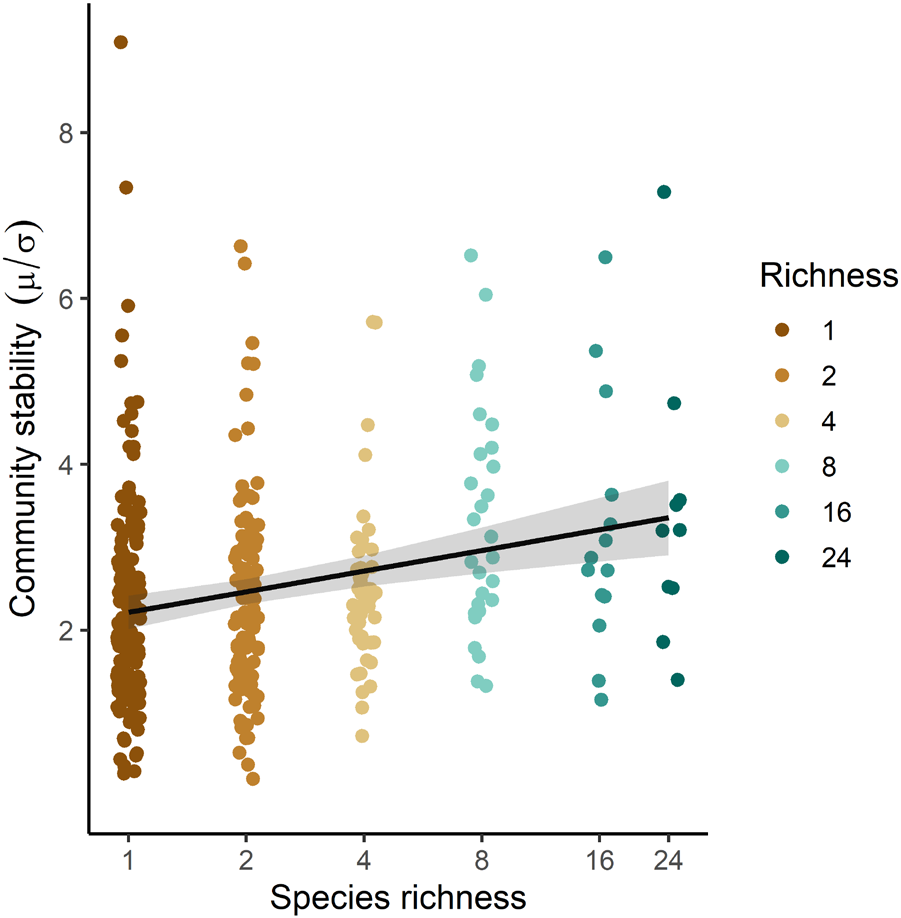
Effects of tree species richness on community stability. The line is a linear mixed-effects model fit that shows a significant increase in community stability with the logarithm of species richness (*P* < 0.001) along a planted diversity gradient ranging from monocultures up to mixtures of 24 tree species. Gray bands represent a 95% confidence interval. See table S2 for details on the fitted model.

**Fig. 3.**
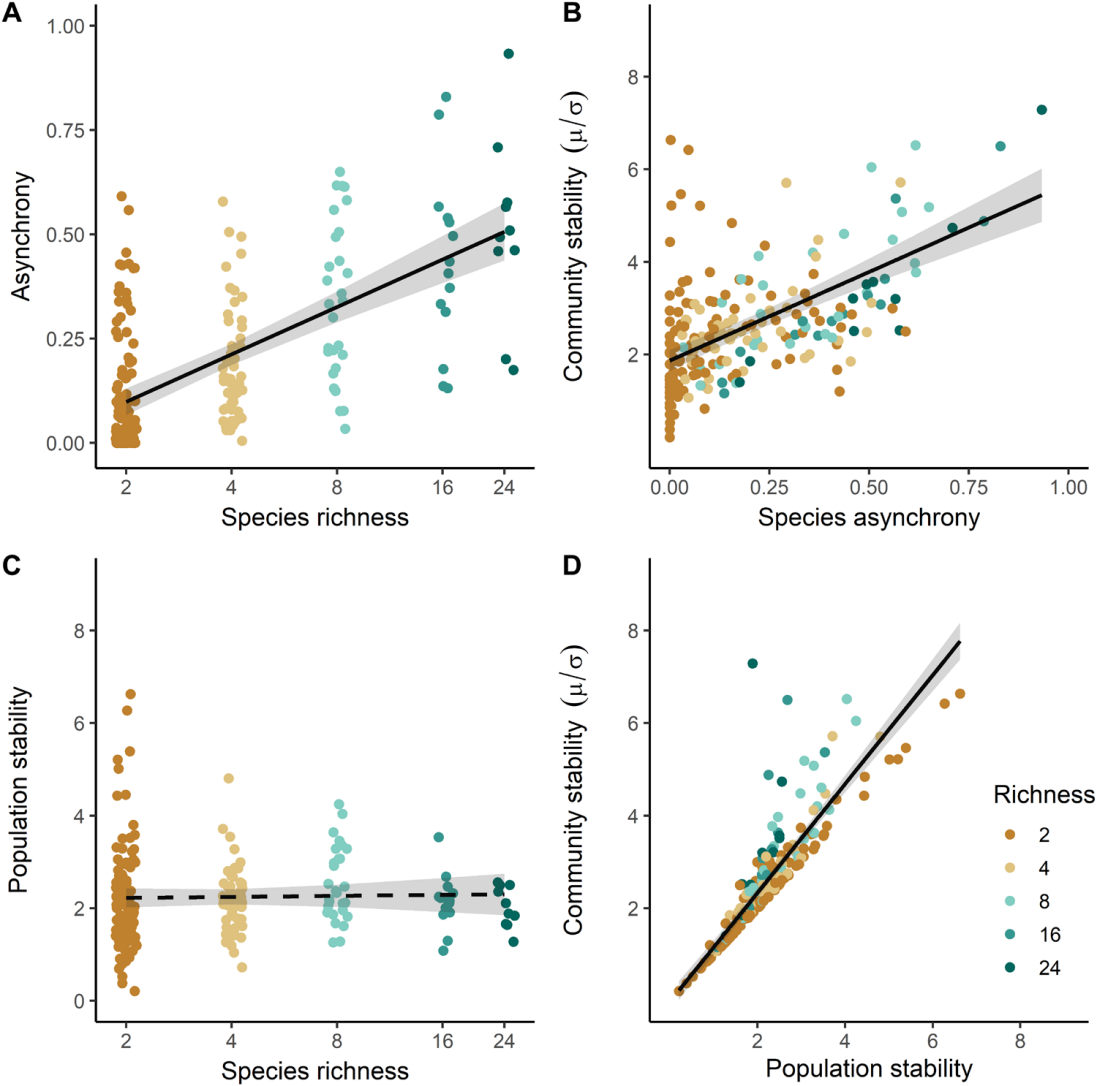
Bivariate relationships between tree species richness, asynchrony, population stability, and community stability. Lines are linear mixed-effects model fits that show (**A**) significant increases in asynchrony with the logarithm of species richness (*P* < 0.001), (**B**) significant increases in community stability with asynchrony (*P* < 0.001), (**C**) no significant relationship between the logarithm of species richness and population stability, and (**D**) significant increases in community stability with population stability (*P* < 0.001). Asynchrony ranges from 0 to 1, where 0 represents complete synchrony and 1 complete asynchrony. Data are based on a long, experimental species richness gradient with mixtures of 2, 4, 8, 16, and 24 tree species. Gray bands represent a 95% confidence interval. See table S2 for details on the fitted models.

Overall, the stability of community productivity significantly increased with species richness in our experimental tree communities (*t* = 3.98, *P* < 0.001, *n* = 375; Fig. 2). This diversity effect was insensitive to the inclusion or exclusion of monocultures into the models (fig. S2). We found a significant increase in asynchrony with species richness (*t* = 9.53, *P* < 0.001) but no effect of species richness on population stability (*t* = 0.27, *P* = 0.785; Fig. 3, A and C, and table S2). Asynchrony and population stability had, as predicted, the strongest positive relationships with community stability in mixtures [*t* = 10.13, *P* < 0.001, marginal coefficient of determination (*R*^2^) = 34% and *t* = 26.30, *P* < 0.001, marginal *R*^2^ = 77%, *n* = 218; Fig. 3, B and D, and table S2]. The relationship between community stability and population stability weakened with increasing asynchrony (fig. S3). We found significant positive relationships between community stability, asynchrony, and drought-tolerance diversity—calculated as functional dispersion in traits related to stomatal control (functional diversity of stomatal control) and in traits related to resistance-acquisition strategies (functional diversity of resistance-acquisition) (Fig. 3B, figs. S4 and S5, and tables S1 and S2). In contrast, the CWMs of these trait gradients did not influence population stability nor community stability (figs. S6 and S7 and table S2). Asynchrony significantly increased with functional diversity of stomatal control (*t* = 5.29, *P* < 0.001) and functional diversity of resistance-acquisition (*t* = 5.84, *P* < 0.001; fig. S4). Relationships of drought-tolerance diversity with community stability were weak: We found a marginally significant positive effect of functional diversity of stomatal control on community stability (*t* = 1.92, *P* = 0.058) but no significant relationship with functional diversity of resistance-acquisition (*t* = 1.12, *P* = 0.27; fig. S5). Drought-tolerance diversity explained a much higher proportion of variability in asynchrony than it did in community stability (table S2). CWMs of drought-tolerance traits did not explain variation in population stability (table S2).

**Fig. 4.**
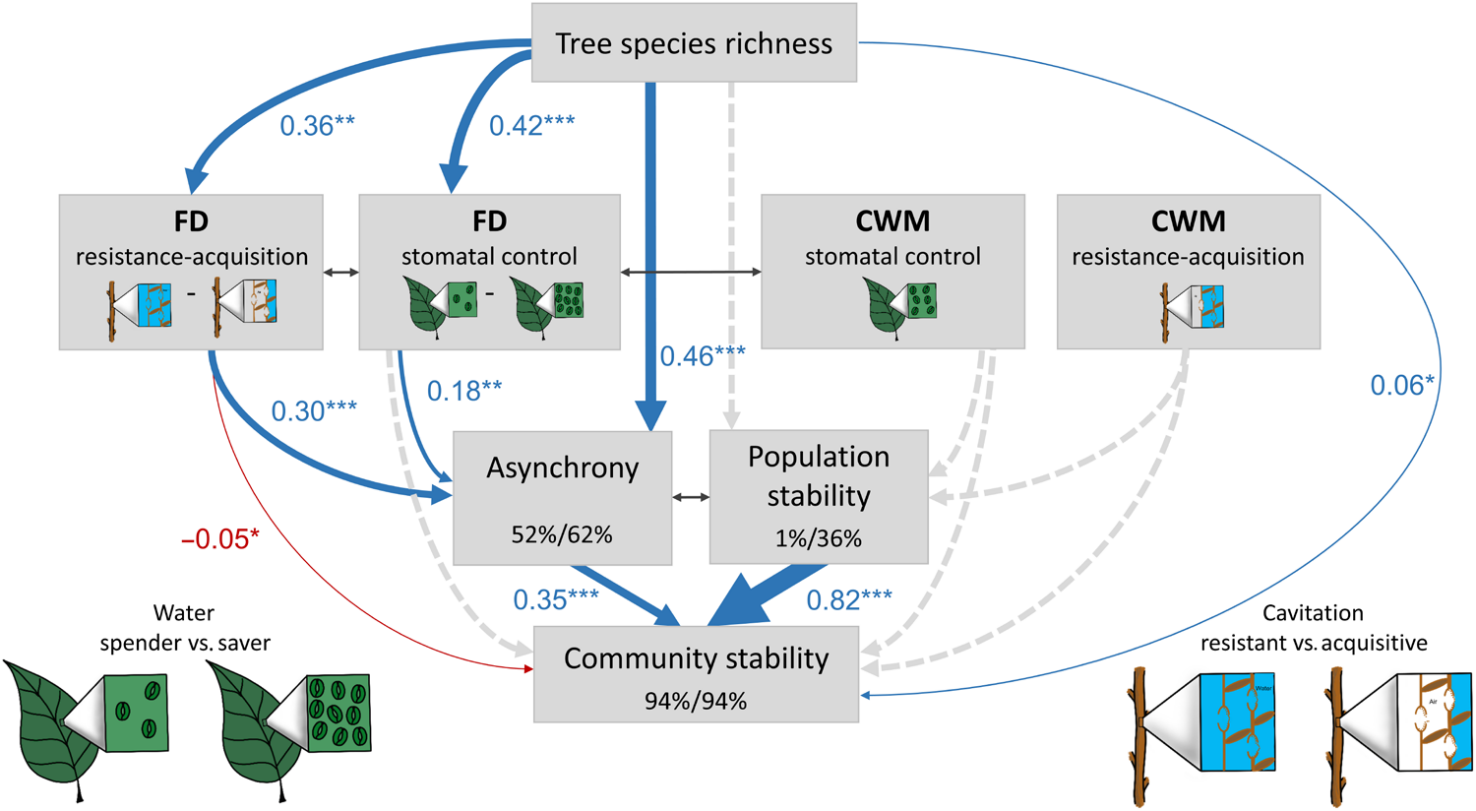
Direct and indirect effects of tree species richness, drought-tolerance diversity, and CWMs of drought-tolerance traits on community stability. The SEM tests the direct effects of tree species richness as well as its indirect effects mediated via asynchrony and population stability on community stability (H1). Effects of functional diversity are explored through testing the effect of functional diversity of stomatal control (FD stomatal control) and functional diversity of resistance-acquisition (FD resistance-acquisition) as well as their indirect effects mediated via asynchrony on community stability (H2). Effects of CWM traits are explored through testing the effect of the CWM of stomatal control (CWM stomatal control) and the CWM of resistance-acquisition (CWM resistance-acquisition) as well as their indirect effects mediated via population stability on community stability (H3). The sketches schematically illustrate the trait gradients: water-spending versus water-saving stomatal control (few versus abundant stomata) and resistant versus acquisitive (high versus low cavitation resistance). Functional diversity was calculated as abundance-weighted functional dispersion. The SEM fit the data well (Fisher’s *C* = 11.7, df = 12, *P* = 0.47, *n* = 218). Data are based on a long, experimental species richness gradient with mixtures of 2, 4, 8, 16, and 24 tree species. Examined variables are shown as boxes and relationships as directional arrows with significant positive effects in blue, significant negative effects in red, and nonsignificant paths in dashed gray based on a hypothesis-driven SEM framework (fig. S8). Standardized (significant) path coefficients are shown next to each path with asterisks indicating significance (*P < 0.05, **P < 0.01, and ***P < 0.001), and path width is scaled by coefficient size. Significant partial correlations (*40*) are shown through gray, bidirectional arrows. Species richness was log_2_-transformed, while asynchrony, population stability, and community stability were square root–transformed. The variation in asynchrony, population stability, and community stability explained by fixed (left, marginal *R*^2^) and fixed together with random model effects (right, conditional *R*^2^) (*71*) is shown in the corresponding boxes.

SEMs allowed us to disentangle the hypothesized direct and indirect drivers and connections behind observed diversity effects on community stability (Fig. 4). Consistent with our hypotheses, asynchrony was the principal mediator of indirect effects of species richness via drought-tolerance diversity on community stability. Our model fit the data well (Fisher’s *C* = 11.7, df = 12, *P* = 0.47, *n* = 218). The hypothesized pathways explained 94% of variation in community stability (fixed effects, marginal *R*^2^). Species richness, functional diversity of stomatal control, and functional diversity of resistance acquisition explained 52% of variation in asynchrony (marginal *R*^2^). In contrast, species richness and the CWMs of drought-tolerance traits explained only 1% of variation in population stability (marginal *R*^2^). Asynchrony and population stability had the strongest direct relationship with community stability (standardized path coefficient of direct effects 0.35, *P* < 0.001 and 0.82, *P* < 0.001, respectively). Tree species richness increased community stability indirectly through increasing asynchrony (standardized path coefficient of direct effect on asynchrony 0.46, *P* < 0.001). Quantifying drought-tolerance diversity allowed us to disentangle some of the potential functional drivers behind asynchronous species responses: Both functional diversity of stomatal control and functional diversity of resistance acquisition were related to increased community stability via positive effects on asynchrony (standardized path coefficients of direct effects on asynchrony 0.18, *P* = 0.005 and 0.30, *P* < 0.001, respectively). Species richness and functional diversity of resistance-acquisition traits had small effects on community stability that canceled each other out (standardized path coefficients of direct effects 0.06 versus −0.05). The strong relationship between population stability and community stability was unrelated to species richness, CWM of stomatal control, and CWM of resistance-acquisition traits (all direct effects on population stability not significant with *P* ≥ 0.45; Fig. 4). Testing asynchrony and population stability in separate SEMs yielded similar results (figs. S9 and S10). In the separate model, we found no remaining direct effect of species richness on stability (*P* = 0.31) after accounting for the pathway via asynchrony, indicating that functional diversity–related asynchrony was the principal mediator of species richness effects on community stability (fig. S9).

**Fig. 5.**
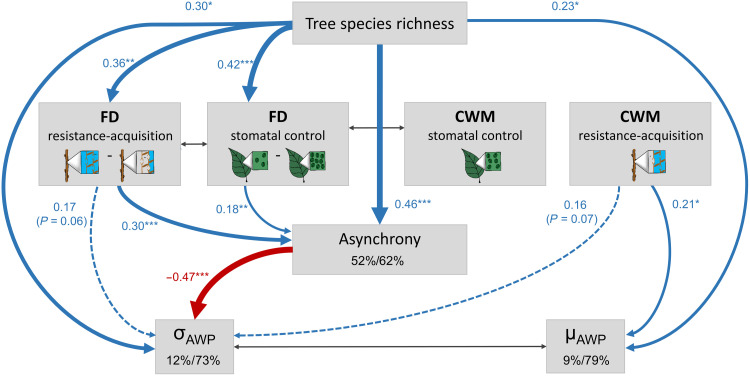
Direct and indirect effects of tree species richness, asynchrony, drought-tolerance diversity, and CWMs of drought-tolerance traits on the two components of stability, the temporal mean (μ_AWP_) and the temporal SD of productivity (σ_AWP_). μ_AWP_ and σ_AWP_ represent overyielding and variance buffering effects, respectively. Increases in μ_AWP_ enhance stability through overyielding—a higher productivity in mixtures versus monocultures—and decreases in σ_AWP_ enhance stability through buffered variations in productivity. All drivers hypothesized to influence stability, i.e., species richness, functional diversity of stomatal control (FD stomatal control), functional diversity of resistance-acquisition (FD resistance-acquisition), CWM of stomatal control (CWM stomatal control), CWM of resistance-acquisition (CWM resistance-acquisition), and asynchrony, were tested for their effects on μ_AWP_ and σ_AWP_. Only significant pathways (*P* < 0.05) are shown here to avoid overplotting (see fig. S11 for the full model). Population stability was not included in this analysis as it did not respond to diversity nor CWM traits (Fig. 4). The sketches schematically illustrate the trait gradients: water-spending versus water-saving stomatal control (few versus abundant stomata) and resistant versus acquisitive (high versus low cavitation resistance). The SEM fit the data well (Fisher’s *C* = 9.7, global *P* = 0.28, df = 8, *n* = 218 plots). Data are based on a long, experimental species richness gradient with mixtures of 2, 4, 8, 16, and 24 tree species. Examined variables are shown as boxes and relationships as directional arrows with significant positive effects in blue, significant negative effects in red, and nonsignificant paths in dashed blue. Standardized (significant) path coefficients are shown next to each path with asterisks indicating significance (*P < 0.05, **P < 0.01, and ***P < 0.001), and path width is scaled by coefficient size. Significant partial correlations (*40*) are shown through gray, bidirectional arrows. Species richness was log_2_-transformed, while asynchrony, μ_AWP_, and σ_AWP_ were square root–transformed. The variation in asynchrony, μ_AWP_, and σ_AWP_ explained by fixed (left, marginal *R*^2^) and fixed together with random model effects (right, conditional *R*^2^) (*71*) is shown in the corresponding boxes.

We further separated the components of our community stability measure—the temporal mean (μ_AWP_) and the temporal SD (σ_AWP_) of productivity—to examine the underlying cause of the observed biodiversity-stability relationships focusing on the role of asynchrony because asynchrony was altered by changes in species richness while population stability was not (Fig. 5). Tree species richness directly increased both the mean and the SD of productivity similarly (standardized path coefficients of direct effects 0.23 and 0.30, respectively). Tree species richness thus increased mean productivity, but this was accompanied by increased variation in productivity. However, species richness also decreased the SD of productivity indirectly via its positive effect on asynchrony with about the same strength [indirect effect of species richness on σ_AWP_ −0.3, calculated as the product of the coefficients along each significant path and their sum Fig. 5; (*40*)]. Asynchrony, which increased with species richness and drought-tolerance diversity, hence stabilized productivity through buffering its temporal variation (standardized path coefficient of direct effect of asynchrony on σ_AWP_ −0.47, *P* < 0.001). Last, the CWM of resistance-acquisition traits was correlated with higher mean productivity and SD of productivity (standardized path coefficients of direct effects 0.21 and 0.16, respectively). That is, communities dominated by acquisitive species with low cavitation resistance (those with higher trait gradient scores; fig. S1) had a higher productivity but tended to also have a higher variation in productivity. Overall, community stability increased with species richness (Fig. 4) through increased mean productivity (i.e., overyielding) and buffered temporal variation in productivity (Fig. 5).

## DISCUSSION

Our results provide experimental evidence that the insurance effect (*14*) of diversity stabilizes tree productivity in forest ecosystems. We show that the stability of forest community productivity increases with tree species richness and that asynchronous productivity of coexisting species is the principal mediator of this diversity effect. Average population stability increased community stability, but this effect was unrelated to species richness. As hypothesized, both functional diversity of stomatal control and functional diversity of resistance-acquisition strategies had net positive, indirect relationships with community stability that operated via positive associations with asynchrony. In contrast, the CWMs of these drought-tolerance traits were not related either to population stability or to community stability.

### Asynchrony, population stability, and community stability

The diversity gradient of the BEF-China experiment (*39*) ranging from monocultures to mixtures of 24 tree species, detailed trait information, and the use of SEMs assessing a priori hypotheses about causal relationships (*40*) based on preexisting knowledge and previous work in this experiment (*36*, *41*) allowed us to disentangle the direct and indirect drivers of community stability in forests in the absence of confounding environmental variation typically hampering interpretations in observational studies. Overall, our models explained higher shares of variation in community stability than did recent work about grassland diversity experiments (*7*) as we accounted not only for the effects of asynchrony but also for those of population stability [94% versus 20% in (*7*)]. Considering only asynchrony (fig. S9), the explained variation in community stability was similar between the two studies (35% versus 20%). We show here that species richness increases community stability indirectly via promoting asynchronous species productivity over time. Community stability and asynchrony were positively correlated with tree species richness in former studies (*5*, *11*–*13*). Our experimental results add support for the hypothesized causality in these studies and demonstrate that species richness can drive asynchrony and thereby community stability in highly diverse subtropical forests. Asynchronous productivity integrates different mechanisms, such as those captured by the selected drought-tolerance traits that help species to cope in different ways with the variable climatic conditions typical for the sites (fig. S12). This asynchrony that was positively related to diverse drought-tolerance strategies enhanced community stability via reducing variation of productivity over the 10-year observation period (*5*, *7*, *12*, *14*, *19*). Species richness also directly increased temporal mean productivity. This finding is in line with a rapidly increasing number of studies reporting that forest productivity increases with increasing tree species richness (*6*, *13*, *16*, *17*). The increased productivity by itself did not increase community stability, because species richness also increased the temporal variation of productivity. Community stability only increased because of the variance buffering effect of asynchrony on productivity (Fig. 5).

The asynchronous growth dynamics of different species in our experimental tree communities likely result from different, nonmutually exclusive mechanisms. First, extrinsic factors like variable climatic conditions may increase asynchrony. Species react differently to climatic conditions [e.g., (*2*, *24*)], and asynchrony is thus likely driven by differential growth responses of species to climatic variability (*25*). Next, tree growth in mixtures is shaped by tree-tree interactions such as resource partitioning and biotic feedbacks (*26*, *27*, *42*, *43*), which may, in turn, be modulated by variation in climatic conditions (*36*, *44*). Last, intrinsic rhythms like mast seeding, which induces interannual variability in species productivity (*22*) and demographic stochasticity (*25*), may influence asynchrony. These factors are, however, presumably less important in young (experimental) forest stands without seedling recruitment or replanting. We thus expect that the observed strong asynchrony resulted predominantly from differential response strategies of species to interannual variation in climatic conditions (the environmental variable with likely the strongest interannual variation during our 10-year study period; fig. S12) and how these strategies, which we quantified via drought-tolerance traits, shape the nature of tree-tree interactions between years with different climatic conditions.

We found that communities dominated by stable growing species populations also had a high community stability, but this effect of population stability was unrelated to species richness. Existing studies, particularly from grasslands, reported a preponderance of negative effects of species richness on population stability due to interspecific competition destabilizing interannual species productivity at higher species richness (*9*, *10*, *12*, *29*). In this view, compensatory population dynamics should decrease population stability but increase community stability via contributing to increased asynchrony (*19*, *28*). However, we did not find significant biodiversity–population stability relationships in our experimental tree communities. This may have different reasons: First, species richness effects on population stability may be smaller in forests than in grasslands as trees invest in long-lasting structures and therefore population dynamics are slower in forests. This may decrease the importance of compensatory dynamics (*5*, *11*). Second, compensatory dynamics may be similarly important in forests, but they need more time to develop or they operate on longer time scales than the 10 years examined in this study. This would be consistent with long-term simulations with a dynamic forest succession model (*12*). Third, beneficial tree-tree interactions (such as facilitation or competitive reduction) during dry years may stabilize species productivity (*5*, *36*), counteracting potential destabilizing effects of interspecific competition. Mirroring this complexity of potential relationships, neutral (*11*), positive (*5*), and negative effects (*12*) of species richness on population stability have been reported for forests. This calls for long-term studies on diversity–population stability relationships particularly in underrepresented ecosystems such as forests. Last, we may have observed stronger positive effects of population stability on community stability when compared to asynchrony (Fig. 3, B and D) and to results from grassland experiments (*45*) because compensatory dynamics are of lower importance in these tree communities. If compensatory dynamics play a minor role in a community, then community stability may have a stronger positive relationship with the intrinsic stability of component species in a community than with asynchrony, as compensatory dynamics should increase asynchrony but decrease population stability (*19*, *28*).

### Drought-tolerance diversity and community stability

We used two orthogonal dimensions in drought-tolerance strategies (fig. S1), related to species-specific stomatal control and resistance-acquisition strategies that have been hypothesized and repeatedly shown to determine forest responses to variable climatic conditions (*2*, *24*, *32*, *36*) and explored their relative contribution to community stability. This allowed us to explain some of the trait-based mechanisms that may be related to asynchronous growth dynamics and stabilize productivity in the face of highly variable climatic conditions. It is important to note that, in the case of traits, we combined our experimental approach of manipulated species richness, with an observational approach as we did not manipulate the mean or variation in drought-tolerance traits nor climatic conditions at our site. Hence, our reported drought tolerance–stability relationships should be considered as correlational evidence until further confirmatory testing is conducted in future experiments that directly manipulate the mean and variation in these traits. Nonetheless, there is accumulating support for the notion that drought-tolerance traits are important for productivity and hence the stability of productivity from former studies in our subtropical tree communities. Comparing 38 traits related to the leaf economics spectrum, stomatal control, leaf and stem hydraulics, and structural and chemical leaf traits, Bongers *et al.* (*41*) found traits related to hydraulic water transport to be the most important and reliable predictors of tree productivity in our experiment. Moreover, Fichtner *et al.* (*36*) showed for the same experiment that annual diversity-productivity relationships were influenced by interannual variation in climate (using the annual climatic water balance shown in fig. S12), that diversity effects were stronger in dry compared to wet years, and that these climate-driven diversity effects were modulated by species drought tolerance [quantified as resistance to cavitation (Ψ_50_), as was done in this study]. These results are consistent with existing evidence that complementary species interactions in forests increase in frequency and intensity with decreasing water availability (*46*). On the basis of these findings, we consider the drought-tolerance traits used here to be suitable traits that may capture interannual changes in productivity as driven by interannual variation in climatic conditions. This is in line with the ubiquity of vulnerability to drought across all forest ecosystems (*2*), including comparably humid subtropical forests. Nonetheless, relationships of leaf or crown area with wood volume (which we used to calculate drought-tolerance diversity and CWMs of drought-tolerance traits) may differ between species and may change plastically along diversity gradients (*47*). Future work would therefore improve our ability to more precisely upscale leaf- and branch-level traits to the community level via characterizing these relationships.

Functional diversity in stomatal control and in resistance-acquisition strategies was positively correlated with increased asynchrony and thus indirectly community stability through reducing variation in productivity. This positive correlation between drought-tolerance diversity and community stability is consistent with recent evidence that tree hydraulic diversity buffers temporal variation in forest ecosystem carbon fluxes during drought (*48*). Functional diversity in stomatal control may promote asynchrony among water spenders and water savers. The former keep their stomata open and continue to transpire during drought. This strategy, however, likely relies on continuous water uptake via roots to balance transpiration losses and carries high cavitation risks (*24*, *32*), a principal mechanism behind drought-induced mortality across tree taxa (*49*). Conversely, water savers can reduce this risk but may face carbon starvation under prolonged droughts (*24*) although starvation is less ubiquitous than cavitation (*49*). These contrasting stomatal-control strategies themselves may induce strong interannual changes in tree growth while also determining the water availability in mixed stands through soil water partitioning between coexisting species (*44*, *50*). In tree neighborhoods comprising species with different stomatal-control strategies, water spenders may benefit from soil water left by their water-saving neighbors during drought, while water savers may capitalize on improved soil water conditions after a drought because of their potentially faster drought recovery (*50*). However, in contrast to resistance-acquisition strategies (see above), there is still little empirical evidence that between-species variation in stomatal-control strategies shapes diversity-productivity relationships during drought.

Functional diversity in resistance-acquisition strategies may promote asynchrony as drought-tolerant species can stabilize the productivity of mixed-species communities through lower risks for xylem cavitation and drought-induced mortality during dry years (*2*, *24*). Conversely, drought-intolerant species, which are characterized by traits associated with an acquisitive resource use strategy in our experiment (see fig. S1) (*33*), can stabilize productivity in wet years. This acquisitive resource use may, moreover, enable soil water partitioning between neighbors during dry years in favor of drought-intolerant species. This could explain why we found acquisitive species to profit most from tree neighborhood diversity during drought in a former study (*36*). Functional diversity in leaf economics spectrum traits is moreover related to diversity in the use of other resources such as light and nutrients (*7*), which may have further contributed to the observed increase in asynchrony and community stability. For example, diversity in shade tolerance quantified via traits of the leaf economics spectrum enhanced asynchrony and thereby stability in simulations with a dynamic forest succession model through both fast responses of shade-intolerant species to forest gaps and a lower susceptibility of shade-tolerant species to disturbances (*12*). Nonetheless, changes in soil nutrients and gap dynamics largely operate on longer time scales than the herein examined 10 years, pointing to a lower importance of nutrient- and light-acquisition strategies for the stability of interannual forest productivity relative to climate variability and water-acquisition strategies. Therefore, and because it would have reduced the strength of our a priori hypotheses, we did not include between-species variation in leaf economics strategies related to light and nutrient acquisition as an additional explanatory variable in our modeling framework. Our analysis focused on between-species variation in drought-tolerance strategies suggests that cavitation resistance and traits of the leaf economics spectrum form a trait syndrome that is related to forest stability. If confirmed in future studies, then this would be an important contribution to the current debate on linkages between drought tolerance and broader dimensions of ecological variation in tropical forest ecosystems [see, for example, Oliveira *et al.* (*37*)].

The direct positive effects of species richness on asynchrony, which remain after accounting for the indirect relationships with drought-tolerance diversity, may result from dissimilarity in traits (*7*) that were not considered here and their potential correlation with the herein examined trait gradients. These traits may include other hydraulically important traits like specific (or maximum) hydraulic conductivity (*K*_s_) (*51*, *52*) as well as other traits not related directly to drought tolerance such as leaf phenology (*23*), storage of nonstructural carbohydrates (*53*), traits regulating biotic feedbacks (*26*), and below- and aboveground structural traits (*47*, *54*, *55*). For example, complementary water uptake through niche differentiation in rooting depth (*56*) and facilitation via hydraulic redistribution (*50*) between species could be important drivers of asynchrony and community stability belowground.

### Community drought-tolerance means and community stability

In contrast to drought-tolerance diversity, the CWMs of drought-tolerance traits did not affect population stability nor community stability. This finding is consistent with a recent analysis of 39 grassland biodiversity experiments where functional diversity but not CWM traits consistently increased community stability across sites (*7*) and with findings from our own site where, after 7 years of stand development, functional diversity and not CWM traits were consistently the stronger and more reliable predictors of forest productivity (*41*). The absence of community mean-trait effects on the stability of community productivity and the preponderance of positive complementarity and negative selection effects developing over time in our experiment (*16*) underline that the observed responses are not simply related to communities becoming increasingly dominated by particularly stable species with stand development. Nonetheless, we found some indication for increased productivity in communities dominated by rather drought-intolerant, acquisitive species, consistent with the common expectation for “fast” growth of these species (*35*, *57*). However, this did not influence community stability because the same communities also had increased variation in productivity. In summary, we found community stability to be positively related to diverse species strategies, such as the here examined diversity in drought-tolerance traits that may help mixed-species tree communities to cope with variable climatic conditions (see Fig. 1) but not to the prevalence of a specific strategy within a community.

### Outlook

The frequency and severity of droughts and corresponding surges in tree mortality are markedly increasing across the globe (*30*, *31*). This situation is expected to worsen with intensifying climate change (*1*), which threatens the climate mitigation potential of the world’s forests (*3*). We show that the stability of forest community productivity along a 10-year observation period increases with tree species richness and that the key driver behind this diversity effect are the asynchronous growth dynamics of different tree species. Community stability did not compromise productivity. Instead, reduced temporal variation in productivity coincided with increased productivity in mixed-species tree communities. Hence, mixing tree species is likely a key management strategy to increase forest community stability and the potential of forests to mitigate the effects of climate change. Drought-tolerance diversity was positively related to community stability via asynchrony suggesting that drought-tolerance traits may be used to select suitable tree species and design mixtures that stabilize productivity in an increasingly variable climate. Here, we examined the stability of young forest communities established as part of a large-scale biodiversity experiment. At the end of the observation period, tree height reached >10 m in 25% of the experimental communities, but reported relationships may differ for older forests. It is conceivable that diversity effects on community stability may strengthen as these stands mature, as indicated by the strengthening diversity effects on productivity (*16*) and by results from an observational study that found stronger positive effects of asynchrony on community stability in old-growth than in secondary forests (*21*). Our results extend research on forest stability from observational studies in relatively species-poor forests (*5*, *11*, *12*) to species-rich subtropical tree communities growing under experimental conditions. This allowed establishing causality and avoiding confounding effects of environmental variation, major issues in observational studies. Community stability increased consistently with tree species richness and did not plateau at low levels of tree species richness, which underlines the enormous potential of species richness to improve forest stability in many of our species-poor or monospecific secondary and plantation forests around the world. This finding has important implications; contemporary forestry, and especially large-scale forest restoration initiatives (*4*), like the Bonn Challenge, should focus on diverse, mixed-species forests to enhance forest stability in a changing climate.

## MATERIALS AND METHODS

### Study site and experimental design

In this study, we used data collected from the BEF-China experiment (www.bef-china.com), located at Xingangshan, Dexing, Jiangxi (29°08′N to 29°11′N, 117°90′E to 117°93′E). BEF-China (*16*, *39*) is a large-scale tree biodiversity experiment that was established at two sites, A and B, each approximately 20 ha in size and planted in 2009 (site A) and 2010 (site B). The study sites are characterized by a subtropical, seasonal monsoon climate with hot and humid summers and dry and cool winters with a mean annual temperature of 16.7°C and mean annual precipitation of 1821 mm (*58*). The sites experienced strong interannual changes in climate-induced water availability during the 10-year observation period (fig. S12), with annual precipitation being more variable than temperature at our study sites (*16*). The highly diverse native subtropical forests of the area are dominated by broadleaved mixed evergreen and deciduous tree species, sometimes interspersed with some conifers (*39*). These forests are located in an area of overlap between tropical and temperate zones (*59*, *60*), which makes them ideally suited to study diverse water use strategies and idiosyncratic species asynchrony as drivers of biodiversity-stability relationships. Furthermore, the region is densely populated and experiences frequent anthropogenic disturbances (*59*), which makes the maintenance and improvement of the functioning of these forests important for the global ecosystem balance and restoration efforts.

The experiment covers a richness gradient ranging from 1 to 24 tree species. Communities have been assembled from a pool of 40 native broadleaved evergreen and deciduous tree species growing in naturally assembled forests of the study region (see table S3 for detailed species information). Species were selected to include a large range of families to maximize functional diversity at higher species richness but without explicitly considering functional diversity as design variable (*39*). To ensure the representation of all species at each diversity level, mixture compositions were randomly allocated following a “broken-stick” design (*39*). In total, 226,400 individual trees were planted on 566 plots (*39*). Dead trees were replanted only during the initial establishment phase until spring 2013. In this study, we used data from six random extinction scenarios allocated to sites A and B (three at each site) with a total of 396 plots and 158,400 planted trees (*16*). Of these, we excluded 21 plots before our analysis because of failed establishment success, which left 375 plots (*n* = 218 mixtures and *n* = 157 monocultures) for our analysis. Each plot had a size of 25.8 × 25.8 m^2^ with 400 individual trees planted in 20 × 20 regular gridded positions (spacing 1.29 m between trees). Tree positions and species compositions were randomly assigned to plots. More detailed information about the BEF-China experiment can be found in the studies by Huang *et al.* (*16*) and Bruelheide *et al.* (*39*).

### Tree data collection

Individual tree basal diameter at 5 cm above ground level (*gd*), tree height, and species identity were measured annually from 2010 (site A) and 2011 (site B) onward at the end of the growing season. To avoid edge effects, the central 12 × 12 trees were measured for each plot in the 4-, 8-, 16-, and 24-species mixtures, while a smaller group of the central 6 × 6 trees was measured for monocultures and 2-species mixtures. Missing tree diameter and height values (in total 2% of census data) were imputed if the increment series was otherwise logical, i.e., value_*t* + 1_ ≥ value_*t* − 1_. To preserve climate-induced growth changes between years during imputation, we used a modeled site-specific rate of growth changes for each yearly step (*r*) based on complete increment series of trees with logical (i.e., with annual increases) and complete census data. A missing tree value was imputed as (*v*_*t* + 1_ − *v*_*t* − 1_) × *r_t_* + *v*_*t* − 1_, where *v* is the *gd* or height measurement in a year, *r* the rate of change, and *t* an index for the year of measurement (see method S1 for details). Overall, we used annual data of 12,852 planted trees from 2010 to 2019 at site A and of 12,204 trees from 2011 to 2019 at site B to estimate community- and species-level productivity.

### Calculation of aboveground wood production

We used aboveground wood volume production as measure of community- and species-level productivity. First, annual aboveground wood volume per tree (*awv*, m^3^) was calculated with a fixed form factor of 0.5 (to account for the noncylindrical shape of trees), which is an average value for the young subtropical trees in our experiment (*43*, *61*); with

awv=ba×h×f (1)

where *ba* is the tree basal area at measured *gd*, *h* the measured tree height, and *f* the form factor. Second, aboveground wood volume production (*awp*, m^3^ year^−1^) per tree and year was calculated as

$$\text{awp}=a{\text{wv}}_{t}-{\text{awv}}_{t-1}$$ (2)

where *t* is an index for the year of measurement. Last, *awv* and *awp* of all trees planted as part of the original design were summed per species and plot and scaled to 1 ha (based on the sampled subplot areas) to derive annual estimates of aboveground wood volume and volume production per species (*AWV_s_*, m^3^ ha^−1^; *AWP_s_*, m^3^ ha^−1^ year^−1^) and community (*AWV*, m^3^ ha^−1^; *AWP*, m^3^ ha^−1^ year^−1^), referred to as species and community productivity. A value of 0 was used in case of species or plots with no alive tree individuals within individual years (note that completely failed plots were excluded from the analysis; see above). Our annual productivity estimates thus cover a complete series of forest growth over the course of 9 and 8 years for sites A and B, respectively.

### Stability and asynchrony of production

The temporal stability (*15*) of tree community productivity, hereafter “community stability,” was calculated as the inverse of the coefficient of variation

$$\text{Stability}=\frac{\mu_{\text{AWP}}}{\sigma_{\text{AWP}}}$$ (3)

where μ*_AWP_* is the temporal mean and σ*_AWP_* the temporal SD of annual plot productivity for our observation period (2010–2019 for site A and 2011–2019 for site B). Thus, any diversity effect that leads to overyielding (a higher productivity of mixtures versus monocultures) increases community stability through increasing temporal mean community productivity μ*_AWP_*. Conversely, any diversity effect that buffers variations in community productivity against changing climatic conditions would increase community stability through decreasing σ*_AWP_* (*14*). We hypothesize here that asynchronous species productivity is the dominant mechanism that stabilizes young tree communities through lowering their community productivity variance. To test this, we calculated community-level species asynchrony (hereafter asynchrony) using the species synchrony statistic φ (*18*) as 1 − φ

$$\text{Asynchrony}=1-\frac{{\sigma_{\text{AWP}}}^{2}}{{\left(\sum_{i=1}^{n}\sigma_{{\text{AWP}}_{s\;i}}\right)}^{2}}$$ (4)

where σ_*AWPs i*_ is the temporal SD of the annual productivity of species *i* in a plot of *n* species (*5*, *62*). Thus, asynchrony increases if the variance in individual species productivity increases relative to the variance in community productivity. Asynchrony ranges from 0 (complete synchrony) to 1 (complete asynchrony) and is, per definition, 0 in monocultures as here variations in community productivity result from variations within a single species (*5*). We expect here that asynchronous species productivity increases community stability through lowering the variation in community-level productivity (see Fig. 1) (*5*). We further hypothesize here that species-level population stability is, next to asynchrony, the second key driver of community stability (*28*). To test this, we calculated average species-level population stability weighted by a species relative abundance (hereafter population stability) as inverse of the average species-level variability measure proposed by Thibaut and Connolly (*28*)

$$\text{Population stability}=\frac{1}{\sum_{i=1}^{n}\frac{\mu_{{\text{AWP}}_{s\;i}}}{\mu_{\text{AWP}}}\times \frac{\sigma_{{\text{AWP}}_{s\;i}}}{\mu_{{\text{AWP}}_{s\;i}}}}=\frac{\mu_{\text{AWP}}}{\sum_{i=1}^{n}\sigma_{{\text{AWP}}_{s\;i}}}$$ (5)

where μ_*AWPs i*_ is the temporal mean of the annual productivity of species *i* in a plot of *n* species. Young tree communities, as the ones examined here, show a strongly increasing productivity over time. As this age trend strongly masks annual variations in productivity, we removed it and calculated community stability as temporal mean productivity divided by its detrended SD. Similarly, asynchrony and population stability were calculated on the basis of detrended plot and species-level productivity. Detrending was performed for each plot and species per plot through regressing annual productivity against time and then calculating the SD on the basis of the residuals of this regression following Craven *et al.* (*7*) and Tilman *et al.* (*9*) (see fig. S13 for a visualization of this approach).

### Trait gradients

Species use different strategies to cope with climate-induced water variability, which are likely related to a set of functional traits related to drought tolerance (*2*, *24*, *32*, *37*, *48*). We assembled species-specific trait data related to stomatal control and cavitation resistance that was measured within the experiment [table S1; (*33*, *34*)]. We focused on these strategies of trees to respond to climate-induced water variability as climate was likely the environmental variable with the strongest interannual variation during the examined 10-year observation period in our experimental tree communities (fig. S12) and as previous studies including two from our experiment suggest a high importance of hydraulic and drought-tolerance traits for tree productivity in general and particularly in response to climate (*24*, *32*, *36*, *41*). Trait data were analyzed with principal components analysis (PCA). The first and second axes partitioned the drought-tolerance traits into two orthogonal trait gradients related to stomatal control (PC1) and cavitation resistance (PC2) (fig. S1). On the basis of physiological and morphological leaf traits, we classified species as water spenders if they decrease their stomatal conductance only at high levels of water pressure deficit, and as water savers, if they already decrease stomatal conductance at low water pressure deficits and have leaves characterized by high stomatal density. We used the water potential at which 50% of xylem conductivity is lost (Ψ_50_) as key physiological trait to quantify a species drought resistance (*2*). Higher values of Ψ_50_ (i.e., lower absolute values of Ψ_50_) indicate a higher susceptibility to drought-induced xylem cavitation. We also included specific leaf area, leaf toughness, and carbon-to-nitrogen ratio as classic traits of the leaf economics spectrum (*35*) in our analysis, as previous studies have shown that these leaf economics spectrum traits are associated with a species cavitation resistance in our study system (*33*, *36*). Hereafter, we refer to this trait gradient therefore as “resistance-acquisition” gradient. We used trait data from 39 of the 40 planted species (*Castanopsis carlesii* was excluded because of complete establishment failure) and imputed two missing trait values (Ψ_50_ and stomatal density) for 1 of these 39 species (*Quercus phillyreoides*) with predicted mean value matching with 500 runs using the R package mice (*63*). PCA was performed with the rda function in the vegan package version 2.5-6 (*64*).

### Quantifying drought-tolerance diversity and community means

We used the scores of the first and second PCA axes (fig. S1) as measure of the species stomatal control and resistance-acquisition strategies within each community. Functional diversity in traits associated with water-spending versus water-saving stomatal behavior (hereafter “functional diversity of stomatal control”) and functional diversity of resistance-acquisition was calculated for each community (plot) with the “FD” package as abundance-weighted functional dispersion (*65*, *66*)

$$\text{FD}=\frac{\sum_{i=1}^{n}a_{i}d_{i}}{\sum_{i=1}^{n}a_{i}}$$ (6)

where *a_i_* is the relative abundance of species *i* in a plot of *n* species, calculated on the basis of temporal mean species wood volume per plot, and *d_i_* is the distance of species *i* to the weighted centroid of the community; see the study by Laliberté and Legendre (*65*) for details. Functional dispersion measures the mean abundance-weighted distance of species along each trait gradient (*65*) and thus represents the potential complementarity in drought-tolerance strategies of co-occurring species within each community. We calculated the CWM trait values for both gradients, hereafter called “CWM of stomatal control” and “CWM of resistance-acquisition” as

$$\text{CWM}=\sum_{i=1}^{n}a_{i}t_{i}$$ (7)

where *t_i_* is the score of species *i* on the respective trait gradient (either stomatal control or resistance-acquisition; fig. S1).

### Modeling framework and statistical analysis

First, we analyzed direct relationships between community stability and its hypothesized drivers and relationships between these drivers. Specifically, we used linear mixed-effects models (LMMs) to test for bivariate relationships between species richness, asynchrony, population stability, functional diversity of stomatal control, functional diversity of resistance-acquisition, CWM of stomatal control, and the CWM of resistance-acquisition. We also tested the effect of species richness and drought-tolerance diversity on asynchrony and the effect of species richness and the CWMs of drought-tolerance traits on population stability. LMMs were fit with the nlme package version 3.1-144 (*67*) to allow for the specification of variance functions with a significance level of α = 0.05. Confidence intervals (95%) of LMM effects were computed with the ggeffects package (*68*). Tree species richness was log_2_-transformed in all models. As the two sites were planted 1 year apart, we tested for a potential age effect and other site-specific influences on the biodiversity-stability relationship through including site and its interaction with species richness as fixed effect. Diversity effects on community stability did not differ between sites (*P* = 0.46 for the interaction). We therefore accounted for site and other aspects of our experimental design through a nested random effect structure of site, species composition, and arrangement of plots within quadrants [see the study by Huang *et al.* (*16*)]. Model assumptions were visually checked for independence and homogeneity of variance through examining model residuals and for normal distribution with quantile-quantile plots. For all response variables, we tested the inclusion of an exponential variance structure (*67*) to model heteroscedasticity [parsimony evaluated via Akaike information criterion (AIC)] and a log/square root transformation to normalize residuals. As results did not differ for any bivariate relationship, we present only the models without variance function or transformation of response variables.

Second, we developed a SEM framework (*40*) to disentangle direct and indirect drivers of community stability based on a priori hypotheses about causal relationships that were informed by preexisting knowledge on mechanism driving biodiversity-stability relationships and by previous work in this experiment (fig. S8). We explored whether the data supported our first hypothesis through including indirect pathways that tested for effects of species richness on community stability that are mediated via asynchrony and population stability (*7*, *28*). We tested our second hypothesis through including indirect pathways that tested for effects of functional diversity of stomatal-control traits and functional diversity of resistance-acquisition traits on community stability through effects mediated via asynchrony (*7*, *36*, *41*). Similarly, we tested our third hypothesis through including indirect pathways that tested for the effects of the CWM of stomatal-control traits and the CWM of resistance-acquisition traits on community stability through effects mediated via population stability (*28*, *36*, *38*, *41*). As the experimental manipulation of species richness may directly affect the functional diversity of a community (*39*), we included pathways from species richness to functional diversity of stomatal control and functional diversity of resistance-acquisition. We further included direct pathways from the diversity facets and the CWMs of both trait gradients to community stability, to test for remaining effects not mediated by asynchrony or population stability. This further allowed us to separately test our second and third hypotheses through either including asynchrony or population stability (figs. S9 and S10). In the absence of population stability, these direct pathways could, for example, account for performance-enhancing effects that increase temporal mean productivity in mixtures (*7*, *13*, *16*), an effect that should otherwise operate via population stability (*28*). Piecewise SEMs (*40*) were used to test the support for and relative importance of these hypothesized pathways. To understand whether observed diversity effects on community stability (Fig. 4) resulted from overyielding (increased μ_AWP_), a buffered variation (decreased σ_AWP_), or both, we fit a separate SEM with these two components of our temporal community stability measure as response. In this second SEM, we tested all hypothesized effects of diversity on community stability for each of its two components (fig. S11). We did not include population stability in this analysis because it did not respond to diversity nor CWM traits.

Global model fit was assessed via Fisher’s *C* statistic (*P* > 0.05). We assessed the independence of variables and included partial, nondirectional correlations if these improved model fit based on tests of directed separations (*P* < 0.05 for violation of independence claims) (*40*). For each SEM, we calculated standardized path coefficients, which allowed us to compare the strength of paths within and among models and of indirect pathways (calculated as product of the coefficients along the path) (*40*). We fitted individual pathways with LMMs using the same random structure and model evaluation as for our analysis of bivariate relationships detailed above. In all SEMs, tree species richness was log_2_-transformed while community stability, asynchrony, population stability, the temporal mean (μ_AWP_), and the temporal SD of productivity (σ_AWP_) were square root–transformed to best meet model assumptions. Our analysis focuses on the drivers of biodiversity-stability relationships. As asynchrony and functional diversity are, per definition, 0 and population stability is equal to community stability in monocultures, we analyzed their effects within 2-, 4-, 8-, 16-, and 24-species mixtures only to avoid many observations without variation. Alternative models including monocultures yielded the same results for effects reported here (figs. S14 and S15). To further test the sensitivity of our models, we ran alternative SEMs without response transformation but with an exponential variance structure for species richness. These yielded the same results (figs. S16 and S17). Last, also the separate test of our second and third hypotheses (figs. S9 and S10) yielded consistent results with our joint SEM model (Fig. 4). SEMs had low variance inflation [variance inflation factor < 5, a conservative threshold choice (*69*)]. All analyses were performed in R 3.6.2 (*70*).

## Supplementary Materials

### This PDF file includes:

Supplementary Text Figs. S1 to S17 Tables S1 to S3 References

## Notes

View/request a protocol for this paper from *Bio-protocol*.
