# Supplementary material for: Species richness stabilizes productivity via asynchrony and drought-tolerance diversity in a large-scale tree biodiversity experiment
Source: Sci Adv. 2021 Dec 17;7(51):eabk1643. doi: 10.1126/sciadv.abk1643 (PMC8682986; doi:10.1126/sciadv.abk1643)
Supplement: Supplementary file 1 — Supplementary Text Figs. S1 to S17 Tables S1 to S3 References [file sciadv.abk1643_sm.pdf]

Science Advances
AAAS

Supplementary Materials for

Species richness stabilizes productivity via asynchrony and drought-tolerance diversity in a large-scale tree biodiversity experiment

Florian Schnabel*, Xiaojuan Liu*, Matthias Kunz*, Kathryn E. Barry, Franca J. Bongers, Helge Bruelheide, Andreas Fichtner, Werner Härdtle, Shan Li, Claas-Thido Pfaff, Bernhard Schmid, Julia A. Schwarz, Zhiyao Tang, Bo Yang, Jürgen Bauhus, Goddert von Oheimb, Keping Ma, Christian Wirth

*Corresponding author. Email: florian.schnabel@idiv.de (F.S.); liuxiaojuan06@ibcas.ac.cn (X.L.); matthias.kunz@tu-dresden.de (M.K.)

Published 17 December 2021, *Sci. Adv.* **7**, eabk1643 (2021)
DOI: 10.1126/sciadv.abk1643

This PDF file includes:

Supplementary Text
Figs. S1 to S17
Tables S1 to S3
References

Supplementary Text

Supplementary Methods 1: Imputation of missing values

Missing diameter or height measurements in individual years are a common challenge in experiments utilizing inventory data sets with a high temporal resolution. Moreover, in 2016 a reduced sample of the central 6×6 trees was measured for all diversity levels except for the very intensively studied mixture plots (see ref (*39*) for details) in contrast to the central 12×12 trees in all other years. We therefore imputed missing tree diameter and height values of trees in a single year caused by forgotten measurements or the reduced sampling size in 2016 as detailed below, but only if the increment series was logical, i.e. $value_{t+1} \geq value_{t-1}$. To ensure that climate induced growth variability between years was preserved during imputation, we calculated site-specific (for site A and B) annual rates of individual tree growth that accounted for observed growth variability between years. We fitted a linear model that predicted annual increment in tree ground diameter (*gd*) by year and *gd* in the preceding year based on measured values of all trees that had complete and completely consistent increment series in all years of our observation period (2009-2019), that is, no missing and always positive increment values. We used the predicted annual increment to calculate rates of relative size change from one year to the next for all years as $r = \frac{i_t}{(i_t + i_{t+1})}$, where i_t is the predicted *gd* increment in year *t*. These annual rates of change were than used to impute each missing measurement as $(v_{t+1} - v_{t-1}) * r_t + v_{t-1}$, where *v* is the *gd* or *height* measurement in a year and *r* the rate of change. In total 2% of values (*gd*, *height* or both) were derived in this way. Hence, our imputation preserved observed annual tree growth changes as driven for example by climatic variability in between years while enhancing the completeness of our annual species and community level productivity estimates per plot.

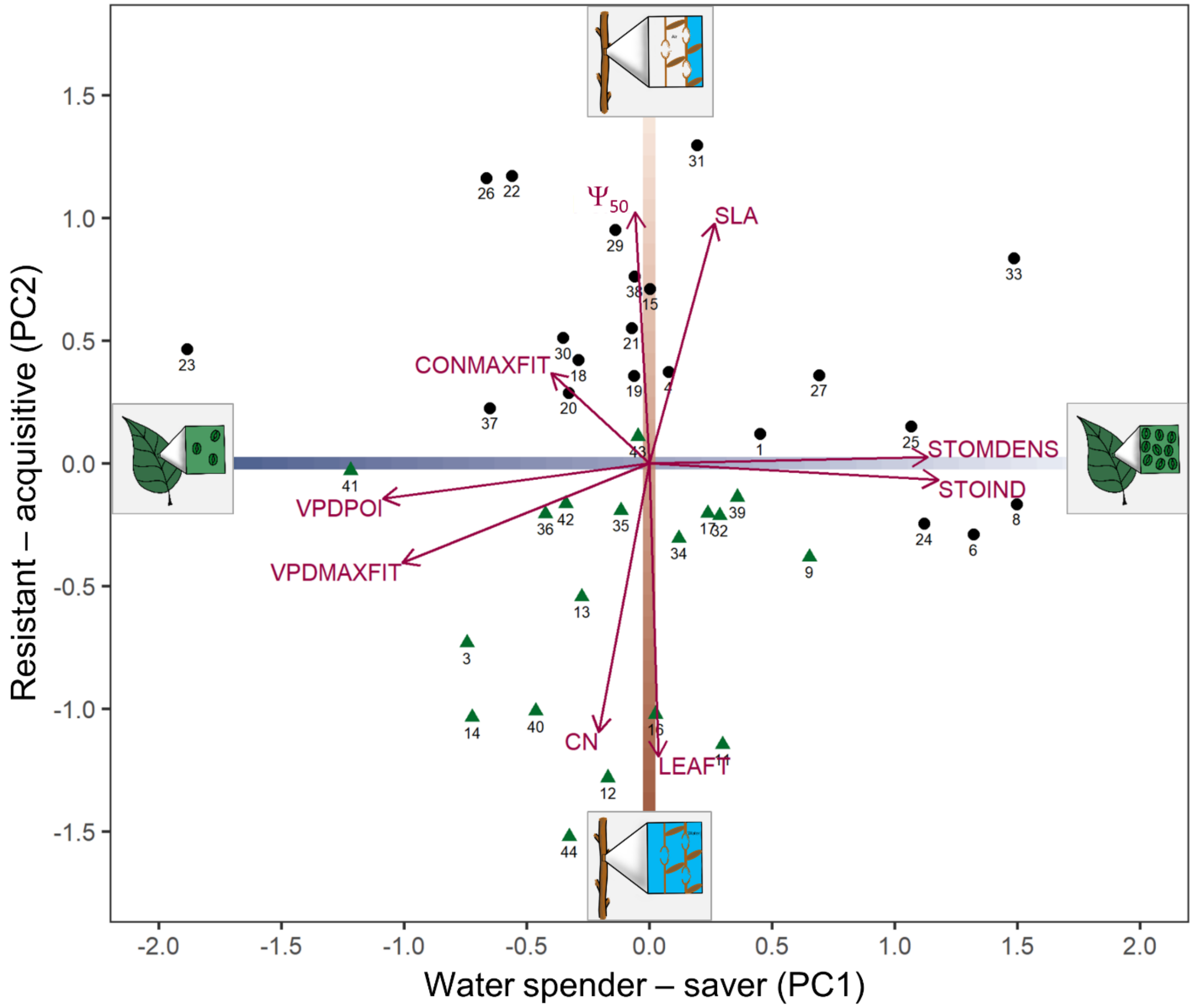

Fig. S1. Principal component analysis (PCA) biplot for the trait-based definition of two drought-tolerance trait gradients. (1) A gradient of stomata control that characterizes species as 'water spenders' if they down-regulate their stomatal conductance only at high levels of measured water pressure deficit (high VPDPOI and VPDMAXFIT), while 'water savers' are species that already down-regulate stomatal conductance at low water pressure deficits and have leaves characterized by a high a stomata density (high STODENS, STOIND; see Table S1 for a trait description). (2) A gradient of drought resistance based on the water potential at which 50% of xylem conductivity is lost (Ψ_{50}) as key physiological trait that expresses a species resistance to water stress (*2*), with less negative values of Ψ_{50} indicating a higher susceptibility to drought induced cavitation. The sketches schematically illustrate the trait gradients: water-spending vs water-saving stomatal control (few versus abundant stomata) and drought resistance (high versus low cavitation resistance). Note, that classic traits of the leaf economics spectrum (LES) (*35*) are associated with cavitation resistance in that species with high cavitation resistance also have traits commonly used to ascribe a conservative resource use strategy (tough leaves (LEAFT) and a high C/N ration (CN)) while low cavitation resistance is associated to a high resource acquisitiveness (high SLA) (*33, 36, 43*). We refer to this trait gradient therefore as 'resistance-acquisition' gradient. All traits (Table S1) were measured on site and used to calculate species level mean trait values, see refs. (*33, 34*) for details. Species identity is shown as species code;

see Table S3 for a detailed species list. Deciduous and evergreen species are coloured as black dots and green triangles, respectively. PCA was performed with the *rda* function in the vegan package version 2.5-6 (*64*). The principal components explained 31% (PC1) and 23% (PC2) of variation. Varimax rotated principal components (base R version 3.6.2, *70*) were used in the analysis to achieve a good alignment of the two orthogonal trait gradients with the first and second PCA axis.

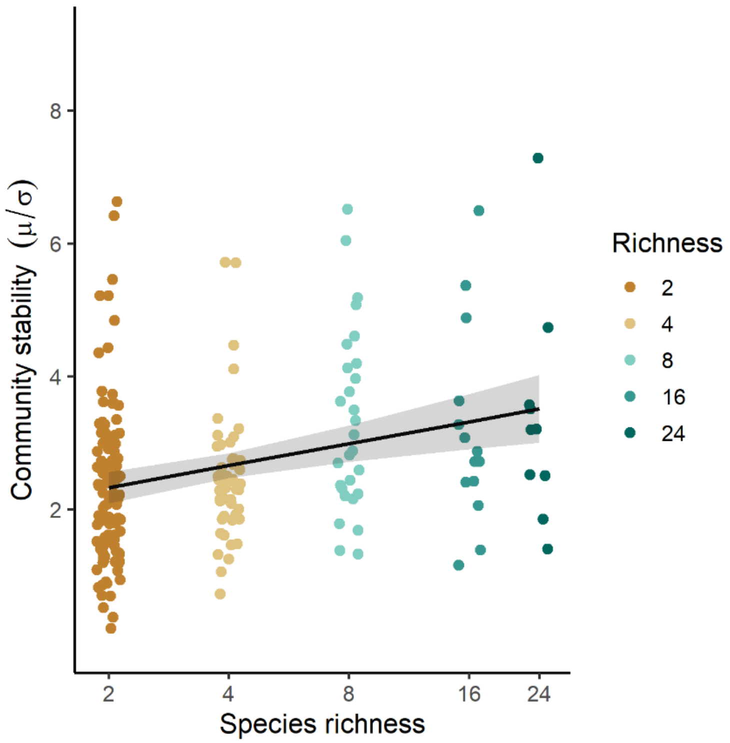

Fig. S2. Effects of species richness on community stability within mixtures. The black line is the fit of a linear mixed-effects model (LMM) that shows a significant increase ($P<0.001$) in community stability with the logarithm of species richness along a planted diversity gradient ranging from 2-species mixtures up to 24-species mixtures (n=218 plots). Grey bands represent a 95% confidence interval. See Table S2 for details on the fitted model. The shown model has a similar model fit compared to the model shown in Fig. 2 which was fit to data from all plots, including monocultures (n=375 plots; see Table S2 for a detailed model comparison).

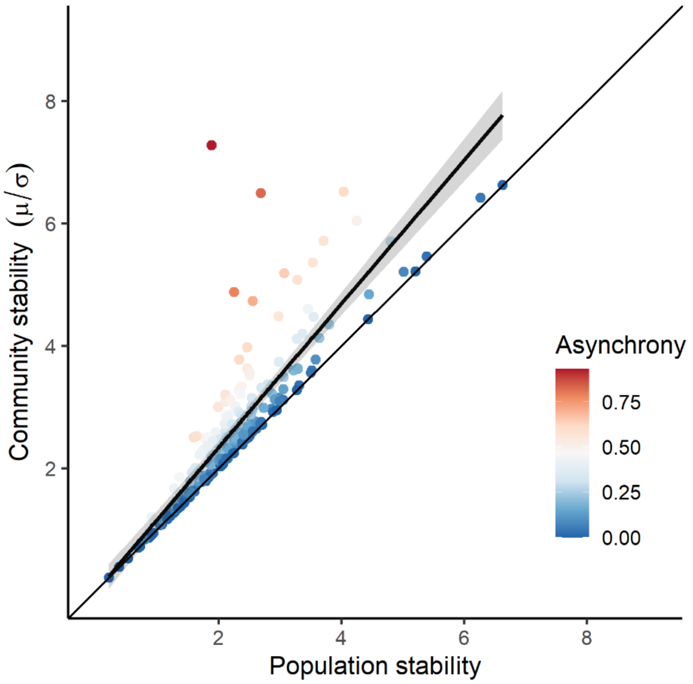

Fig. S3. Effects of population stability on community stability with increasing asynchrony. The line is a linear mixed-effects model fit that shows a significant increase in community stability with population stability in mixtures ($P<0.001$). The thin 1-1 line shows where community stability is equal to average species-level population stability. Points are coloured according to their value with deeper red indicating increasing asynchrony. Grey bands represent a 95% confidence interval. See Table S2 for details on the fitted model. The model is the same as the one shown in Fig. 3D.

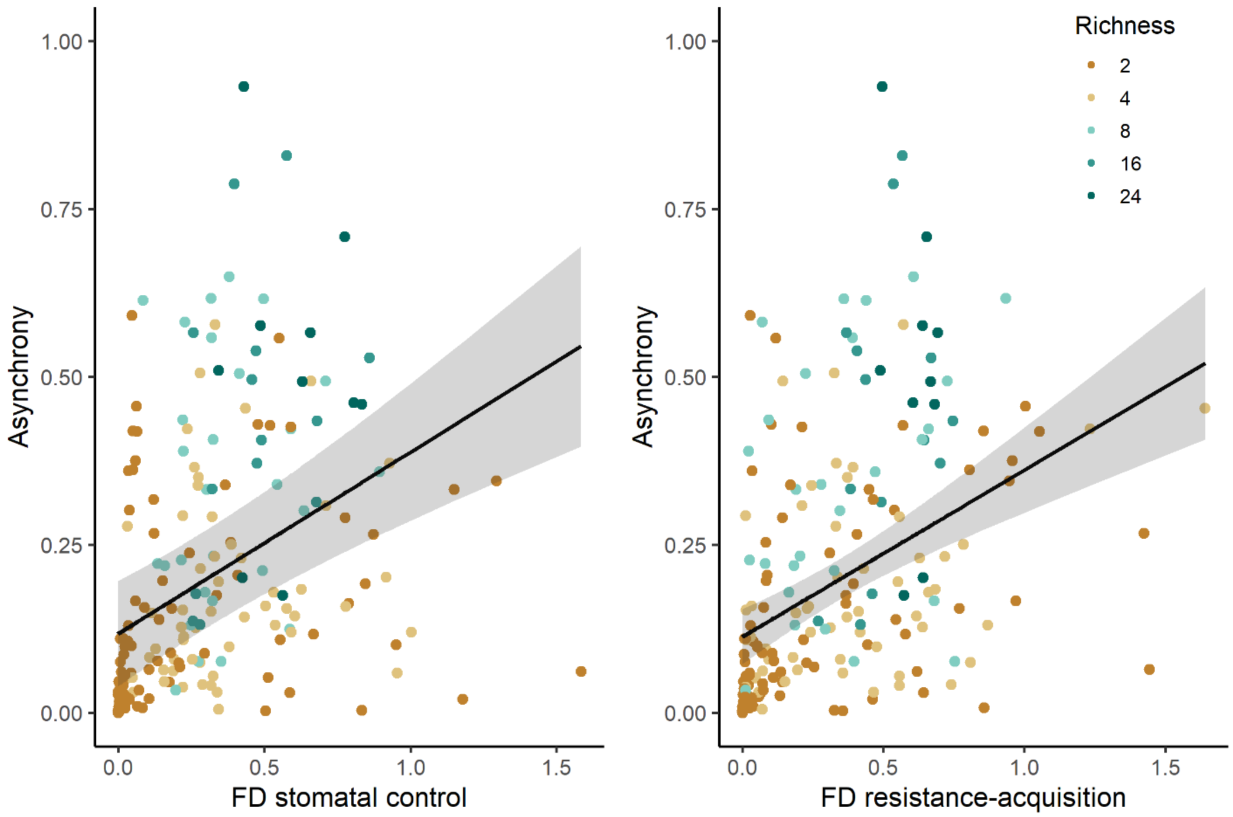

Fig. S4. Relationships between drought-tolerance diversity and asynchrony. Lines are linear mixed-effects model fits that show (a) significant increases in asynchrony with functional diversity of stomatal control (FD stomatal control; $P<0.001$) and (b) significant increases in asynchrony with functional diversity of resistance-acquisition (FD resistance-acquisition; $P<0.001$) in mixtures. Asynchrony ranges from 0 to 1. 0 represents complete synchrony and 1 represents complete asynchrony. Functional diversity calculated as abundance-weighted functional dispersion. Grey bands represent a 95% confidence interval. See Table S2 for details on the fitted models.

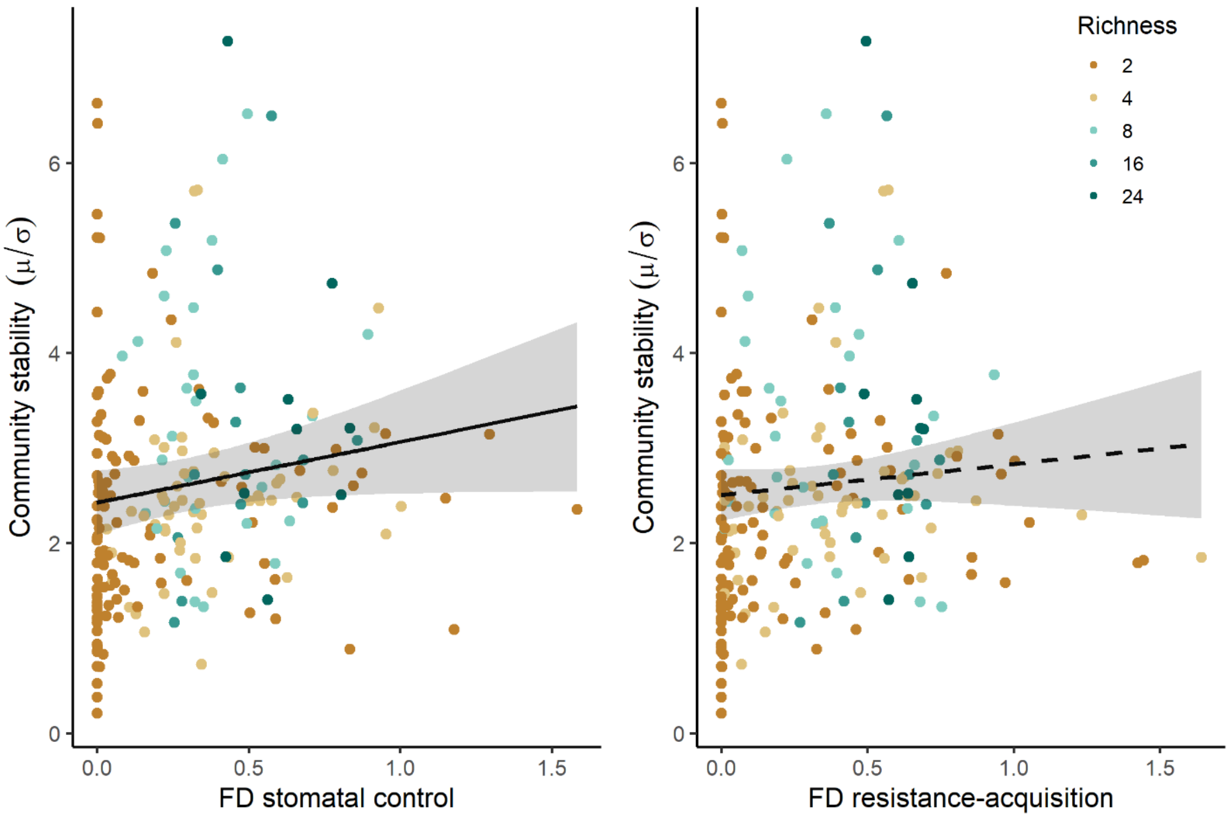

Fig. S5. Relationships between drought-tolerance diversity and community stability. Lines are linear mixed-effects model fits that show (a) a marginally significant increase in community stability with functional diversity of stomatal control (FD stomatal control; P=0.058) and (b) a non-significant relationship of community stability with functional diversity of resistance-acquisition (FD resistance-acquisition; P=0.27) in mixtures. Functional diversity calculated as abundance-weighted functional dispersion. Grey bands represent a 95% confidence interval. See Table S2 for details on the fitted models.

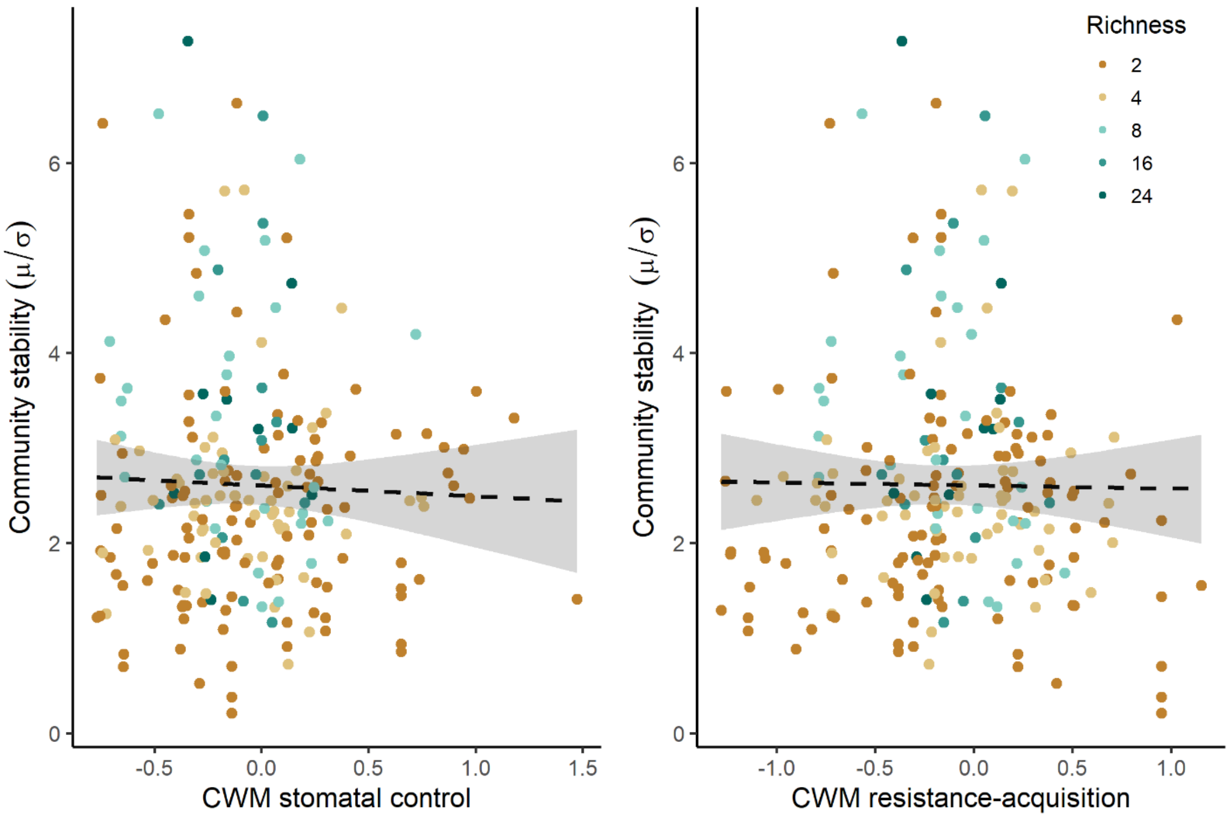

Fig. S6. Relationships between CWMs of drought-tolerance traits and community stability. Lines are linear mixed-effects model fits that show (a) a non-significant relationship of community stability with the CWM of stomatal control (CWM stomatal control; P=0.65) and (b) a non-significant relationship of community stability with the CWM of resistance-acquisition (CWM resistance-acquisition; P=0.88) in mixtures. Grey bands represent a 95% confidence interval. See Table S2 for details on the fitted models.

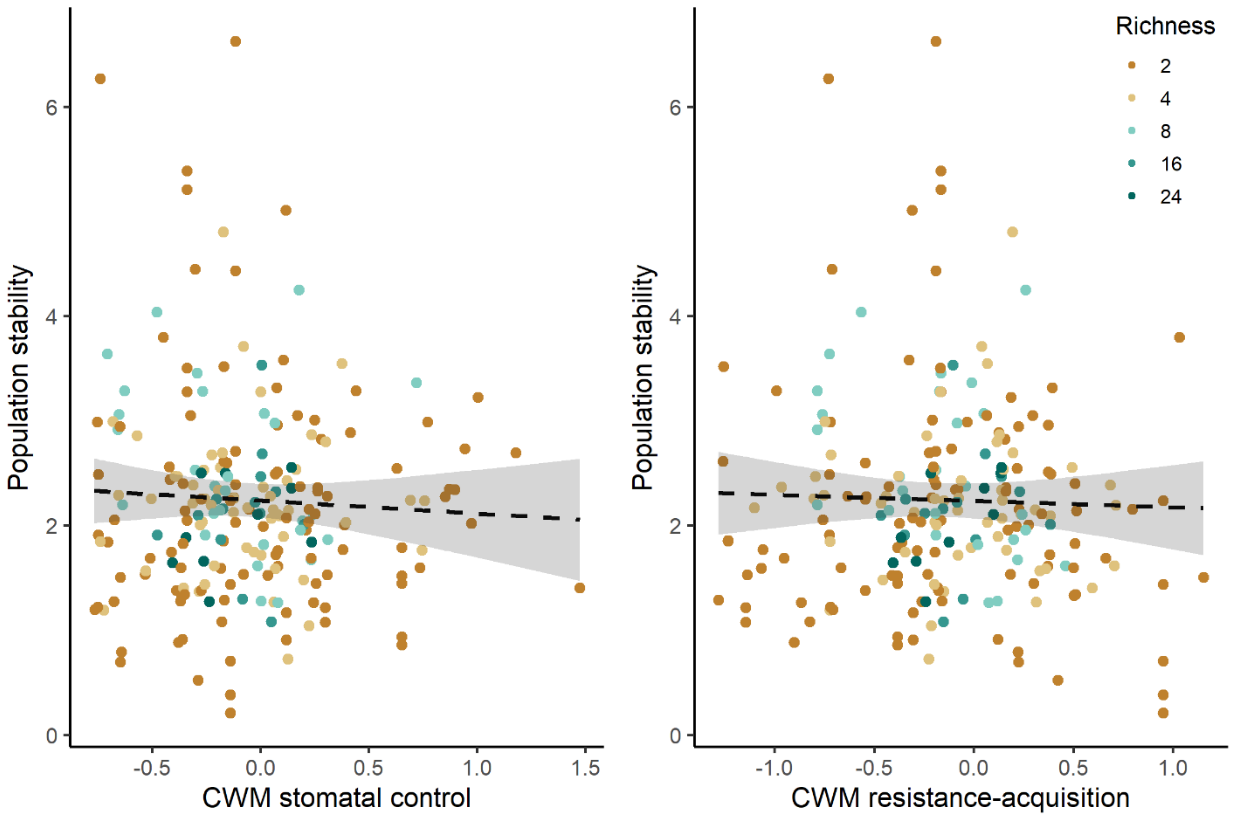

Fig. S7. Relationships between CWMs of drought-tolerance traits and population stability. Lines are linear mixed-effects model fits that show (a) a non-significant relationship of population stability with the CWM of stomatal control (CWM stomatal control; P=0.52) and (b) a non-significant relationship of population stability with the CWM of resistance-acquisition (CWM resistance-acquisition; P=0.72) in mixtures. Grey bands represent a 95% confidence interval. See Table S2 for details on the fitted models.

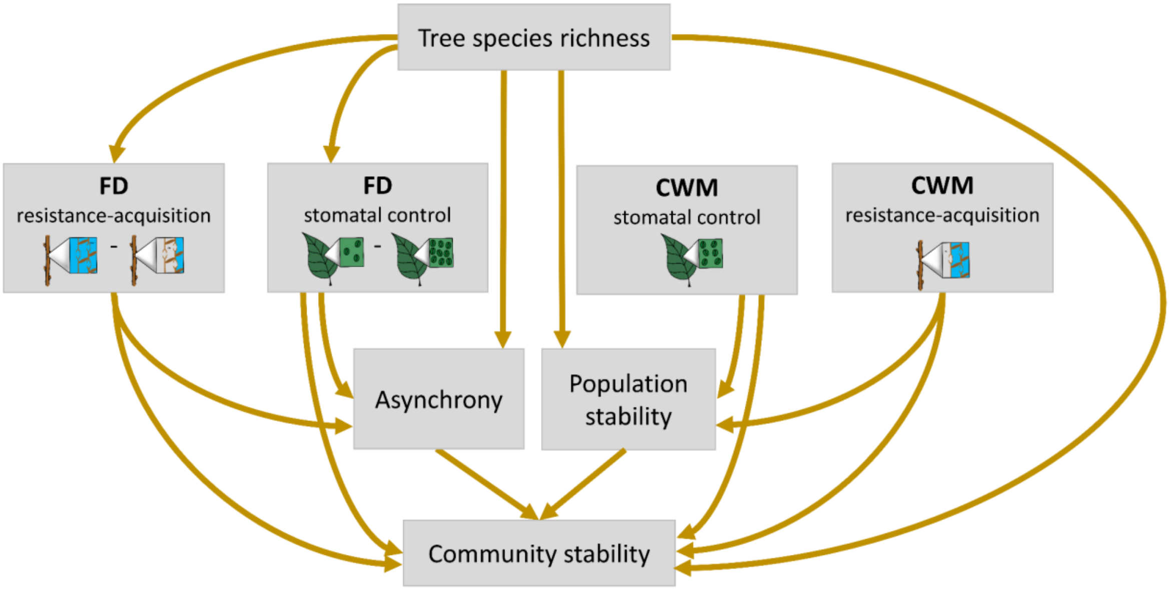

Fig. S8. Hypotheses driven framework of the direct and indirect drivers of community stability in mixed-species tree communities. Directional arrows represent hypothesized causal relationships. We explored whether the data supported our first hypothesis through including indirect pathways that tested for effects of species richness on community stability that are mediated via asynchrony and population stability (7, 28). We tested our second hypothesis through including indirect pathways that tested for effects of functional diversity of stomatal-control traits and functional diversity of resistance-acquisition traits on community stability through effects mediated via asynchrony (7, 36, 41). Similarly, we tested our third hypothesis through including indirect pathways that tested for the effects of the CWM of stomatal-control traits and the CWM of resistance-acquisition traits on community stability through effects mediated via population stability (28, 36, 38, 41). As the experimental manipulation of species richness may directly affect the functional diversity of a community (39), we included pathways from species richness to functional diversity of stomatal control and functional diversity of resistance-acquisition. We further included direct pathways from the diversity facets and the CWMs of both trait gradients to community stability, to test for remaining effects not mediated by asynchrony or population stability. This further allowed us to test our second and third hypothesis separately through either including asynchrony or population stability (Figs. S9–S10). In the absence of population stability, these direct pathways could for example account for performance enhancing effects that increase temporal mean productivity in mixtures (7, 13, 16), an effect that should otherwise operate via population stability (28). The sketches schematically illustrate the trait gradients: water-spending vs water-saving stomatal control (few versus abundant stomata) and resistant vs acquisitive (high versus low cavitation resistance). This framework was tested with data from experimental tree communities from the BEF-China experiment (*16*, *39*), that span a long gradient of planted tree species richness with mixtures of up to 24 different tree species using piecewise structural equation models (SEMs) (*40*).

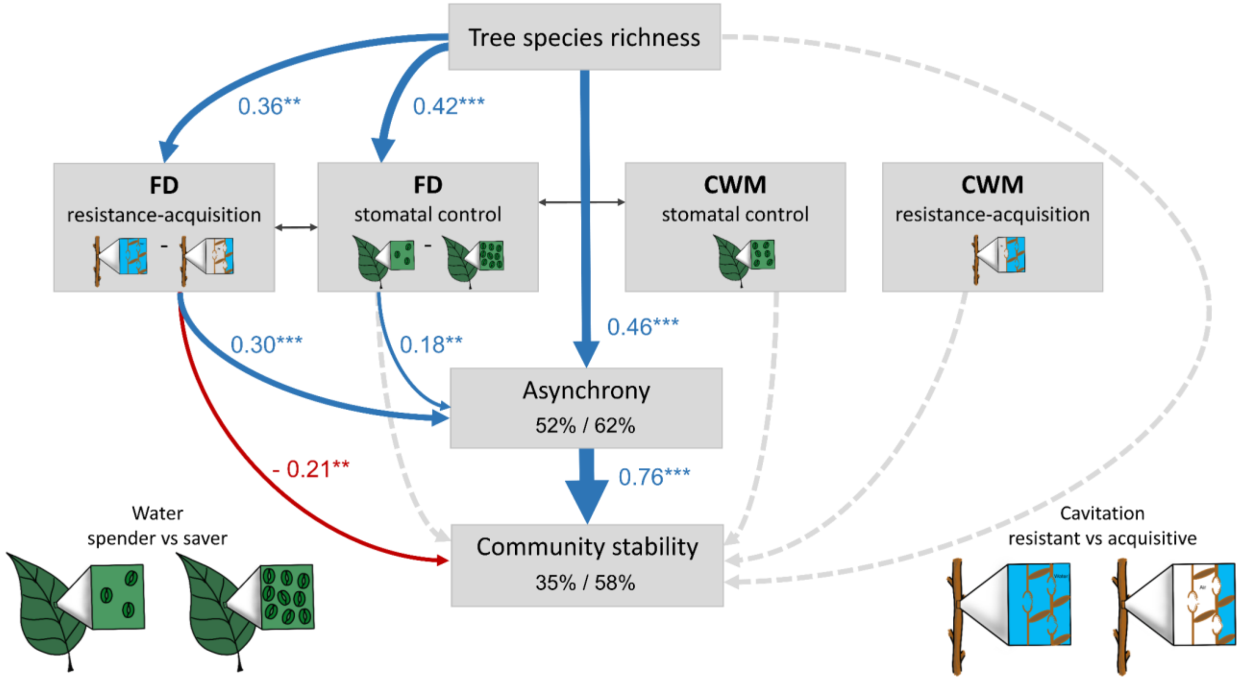

Fig. S9 Direct and indirect effects of tree species richness, drought-tolerance diversity and CWMs of drought-tolerance traits on community stability. The structural equation model (SEM) tests the direct effects of tree species richness, functional diversity of stomatal control (FD stomatal control) and functional diversity of resistance-acquisition (FD resistance-acquisition) as well as their indirect effects mediated via asynchrony on community stability in the absence of population stability. Effects of CWM traits are explored through testing the effect of the CWM of stomatal control (CWM stomatal control) and the CWM of resistance-acquisition (CWM resistance-acquisition) on community stability. The sketches schematically illustrate the trait gradients: water-spending vs water-saving stomatal control (few vs abundant stomata) and resistant vs acquisitive (high versus low cavitation resistance). Functional diversity was calculated as abundance-weighted functional dispersion. The SEM fit the data well (Fisher's C=9.7, P =0.28, d.f.=8, n=218 plots). Data is based on a long, experimental species richness gradient with mixtures of 2, 4, 8, 16 and 24 tree species. Examined variables are shown as boxes and relationships as directional arrows with significant positive effects in blue, significant negative effects in red and non-significant paths in dotted grey based on a hypothesis driven SEM framework (Fig. S8). Standardized (significant) path-coefficients are shown next to each path with asterisks indicating significance (* P<0.05, ** P<0.01, *** P<0.001), path-width is scaled by coefficient size. Significant partial correlations are shown through grey, bi-directional arrows. Species richness was $\log_2$ transformed while asynchrony and community stability were square-root transformed. The variation in asynchrony and community stability explained by fixed (left, marginal R^2) and fixed together with random model effects (right, conditional R^2) is shown in the corresponding boxes. Note that the unexpected direct negative effect of functional diversity of drought tolerance on stability can be attributed to its positive effect on the temporal standard deviation (Fig. 5, marginally significant, P=0.06). This could be because the likelihood of including highly drought sensitive species increases with increasing tree species richness. Increased mortality as a result of including these sensitive species may cause increased temporal variation in community productivity at high species richness, as observed here (see Fig. 5).

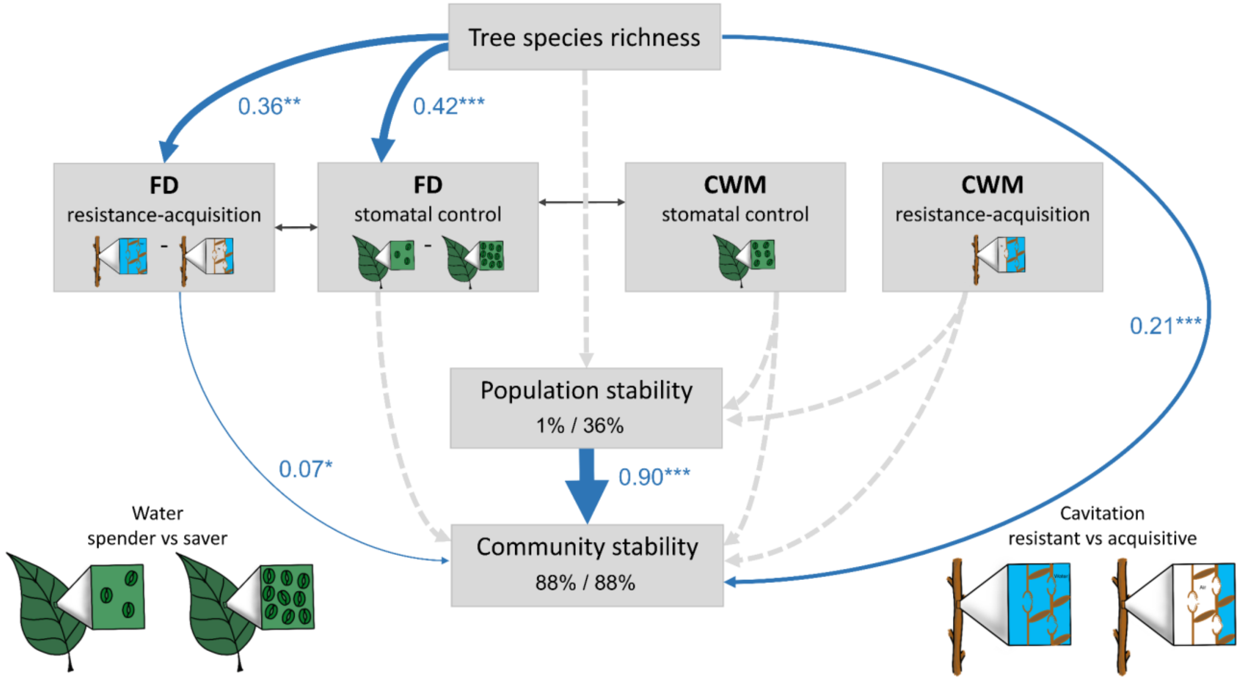

Fig. S10 Direct and indirect effects of tree species richness, CWMs of drought-tolerance traits and drought-tolerance diversity on community stability. The structural equation model (SEM) tests the direct effects of tree species richness, the CWM of stomatal control (CWM stomatal control) and the CWM of resistance-acquisition (CWM resistance-acquisition) as well as their indirect effects mediated via population stability on community stability in the absence of asynchrony. Effects of drought-tolerance diversity are explored through testing the effect of stomatal control (FD stomatal control) and functional diversity of resistance-acquisition (FD resistance-acquisition) on community stability. The sketches schematically illustrate the trait gradients: water-spending vs water-saving stomatal control (few vs abundant stomata) and resistant vs acquisitive (high versus low cavitation resistance). Functional diversity was calculated as abundance-weighted functional dispersion. The SEM fit the data well (Fisher's C=5.2, P =0.73, d.f.=8, n=218 plots). Data is based on a long, experimental species richness gradient with mixtures of 2, 4, 8, 16 and 24 tree species. Examined variables are shown as boxes and relationships as directional arrows with significant positive effects in blue, significant negative effects in red and non-significant paths in dotted grey based on a hypothesis driven SEM framework (Fig. S8). Standardized (significant) path-coefficients are shown next to each path with asterisks indicating significance (* P<0.05, ** P<0.01, *** P<0.001), path-width is scaled by coefficient size. Significant partial correlations are shown through grey, bi-directional arrows. Species richness was $\log_2$ transformed while population stability and community stability were square-root transformed. The variation in population stability and community stability explained by fixed (left, marginal R^2) and fixed together with random model effects (right, conditional R^2) is shown in the corresponding boxes.

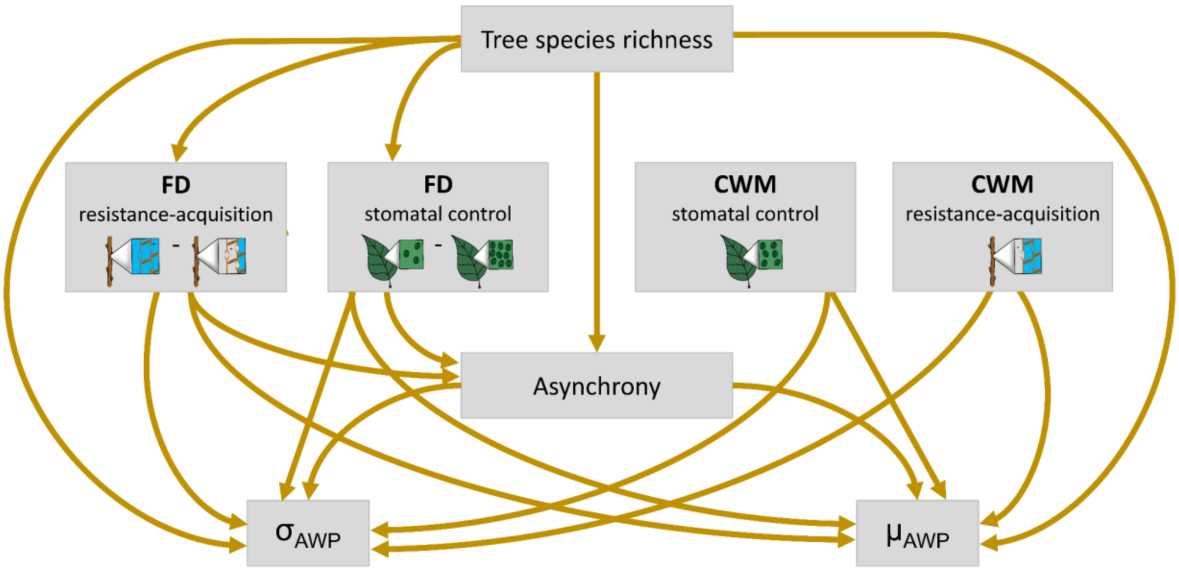

Fig. S11. Hypotheses driven framework for the partitioning of the direct and indirect effects of species richness, drought-tolerance diversity and CWMs of drought-tolerance traits into overyielding and variance buffering effects of diversity. Directional arrows represent hypothesized pathways. The framework separates the hypothesized effects of diversity on community stability (Fig. S8) into effects on the temporal mean (μ_{AWP}) and the temporal standard deviation of productivity (σ_{AWP}). Increases in μ_{AWP} would enhance community stability through overyielding — a higher productivity in mixtures vs monocultures — and decreases in σ_{AWP} would enhance community stability through buffered variations in productivity. All drivers hypothesized to influence community stability (Fig. S8) – species richness, functional diversity of stomatal control (FD stomatal control), functional diversity of resistance-acquisition (FD resistance-acquisition), the CWM of stomatal control (CWM stomatal control), the CWM of resistance-acquisition (CWM resistance-acquisition) and asynchrony – are partitioned here into their effects on μ_{AWP} and σ_{AWP}. Population stability was not included in this framework, as it did not respond to diversity nor CWM traits (Fig. 4). The sketches schematically illustrate the trait gradients: water-spending vs water-saving stomatal control (few versus abundant stomata) and resistant vs acquisitive (high versus low cavitation resistance). This framework was tested with data from experimental tree communities from the BEF-China experiment (*16*, *39*), that span a long gradient of planted tree species richness with mixtures of up to 24 different tree species using piecewise structural equation models (SEMs) (*40*).

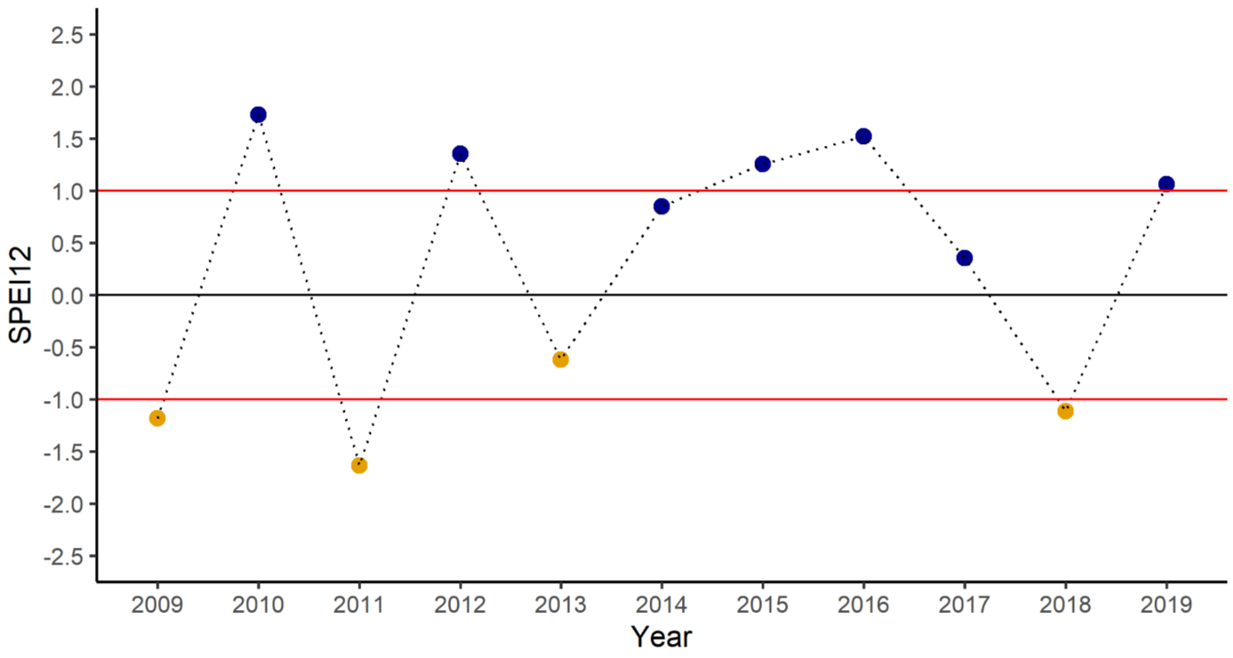

Fig. S12. Annual values of the Standardised Precipitation-Evapotranspiration Index (SPEI). The drought index (*72*) captures the annual standardized water balance (precipitation minus potential evapotranspiration) and its variation during our study period. Negative values indicate climatic water deficits (brown coloured points) and positive values a water surplus (blue coloured points). Years with values above 1 or below -1 can be considered exceptionally wet and dry, respectively. The SPEI index was calculated based on monthly resolved precipitation and potential evapotranspiration data (CRU TS 4.04) (*73*) and is expressed here as annual water balance between two tree census intervals (SPEI12, water balance from September to October of the preceding year). The SPEI index was calculated with the SPEI package (*74*) in R using climate data from 1901–2019 as a reference period.

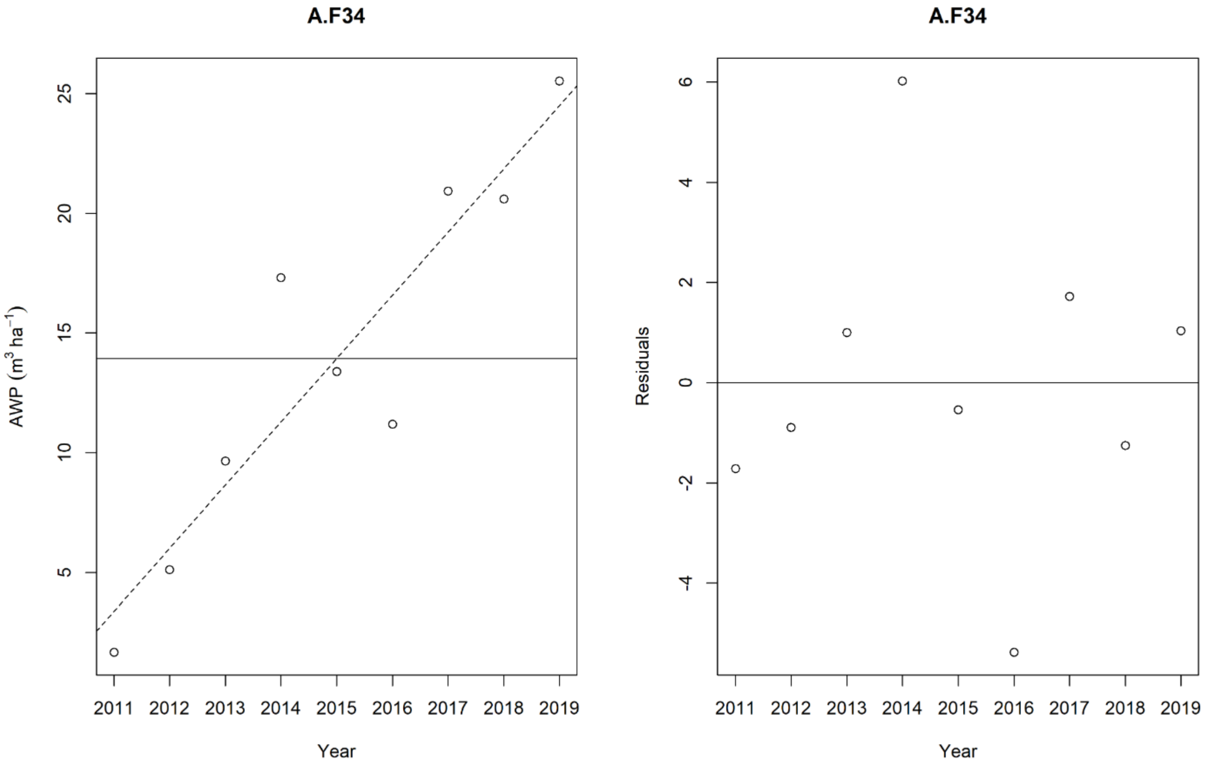

Fig. S13. Example for the calculation of detrended community stability. (A) Shows annual aboveground wood volume productivity (AWP, from plot F34 on site A) regressed against time. (B) The residuals of this regression represent the annual variation in productivity without a directional stand development trend. The standard deviation of these residuals gives the detrended standard deviation that was subsequently used to calculate detrended community stability as temporal mean productivity (μ_{AWP}) divided by its detrended standard deviation (σ_{AWP}).

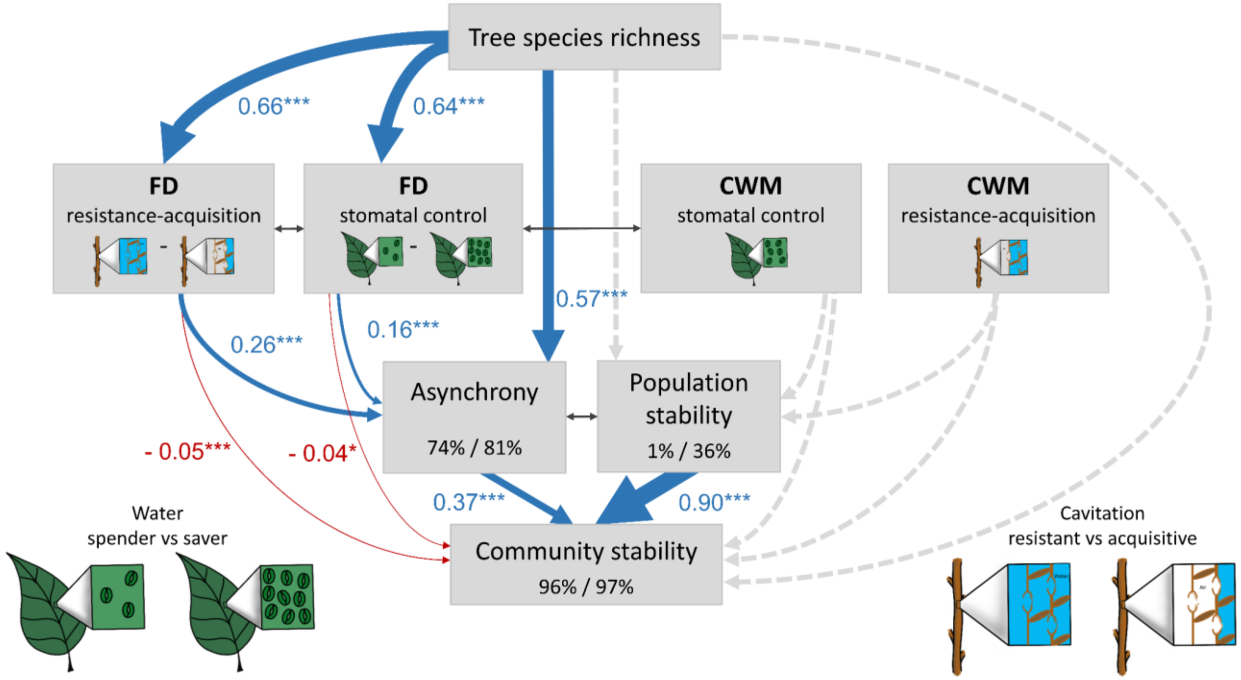

Fig. S14. Direct and indirect effects of tree species richness, drought-tolerance diversity and CWMs of drought-tolerance traits on community stability. The structural equation model (SEM) is fit to data including monocultures and tests the direct effects of tree species richness as well as its indirect effects mediated via asynchrony and population stability on community stability (H1). Effects of functional diversity are explored through testing the effect of functional diversity of stomatal control (FD stomatal control) and functional diversity of resistance-acquisition (FD resistance-acquisition) as well as their indirect effects mediated via asynchrony on community stability (H2). Effects of CWM traits are explored through testing the effect of the CWM of stomatal control (CWM stomatal control) and the CWM of resistance-acquisition (CWM resistance-acquisition) as well as their indirect effects mediated via population stability on community stability (H3). The sketches schematically illustrate the trait gradients: water-spending vs water-saving stomatal control (few versus abundant stomata) and resistant vs acquisitive (high versus low cavitation resistance). Functional diversity was calculated as abundance-weighted functional dispersion. The SEM fit the data well (Fisher's C=11.6, P=0.48, d.f.=12, n=375 plots). Data is based on a long, experimental species richness gradient ranging from monocultures to mixtures of 24 tree species. Examined variables are shown as boxes and relationships as directional arrows with significant positive effects in blue, significant negative effects in red and non-significant paths in dotted grey based on a hypothesis driven SEM framework (Fig. S8). Standardized (significant) path-coefficients are shown next to each path with asterisks indicating significance (* P<0.05, ** P<0.01, *** P<0.001), path-width is scaled by coefficient size. Significant partial correlations are shown through grey, bi-directional arrows. Species richness was $\log_2$ transformed while asynchrony, population stability and community stability were square-root transformed. The variation in asynchrony and community stability explained by fixed (left, marginal R^2) and fixed together with random model effects (right, conditional R^2) is shown in the corresponding boxes.

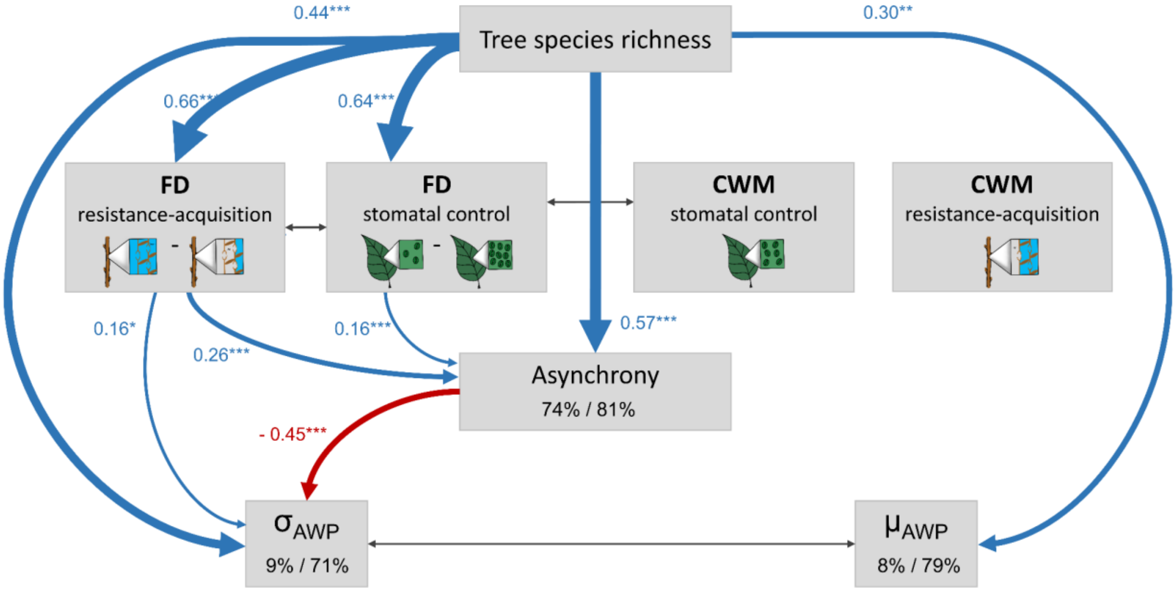

Fig. S15. Partitioning of the direct and indirect effects of species richness, drought-tolerance diversity and CWMs of drought-tolerance traits into overyielding and variance buffering effects of diversity. The structural equation model (SEM) is fit to data including monocultures and separates the hypothesized effects of diversity on community stability (Fig. S8) into effects on the temporal mean (μ_{AWP}) and the temporal standard deviation of productivity (σ_{AWP}). Increases in μ_{AWP} enhance community stability through overyielding – a higher productivity in mixtures vs monocultures - and decreases in σ_{AWP} enhance community stability through buffered variations in productivity. All drivers hypothesized to influence community stability – species richness, functional diversity of stomatal control (FD stomatal control), functional diversity of resistance-acquisition (FD resistance-acquisition), the CWM of stomatal control (CWM stomatal control), the CWM of resistance-acquisition (CWM resistance-acquisition) and asynchrony - were tested for their effects on μ_{AWP} and σ_{AWP}. Only significant pathways (P<0.05) are shown here to avoid overplotting (see Fig. S11 for the full model). Population stability was not included in this analysis, as it did not respond to diversity nor CWM traits (Fig. 4). The sketches schematically illustrate the trait gradients: water-spending vs water-saving stomatal control (few versus abundant stomata) and resistant vs acquisitive (high versus low cavitation resistance). The SEM fit the data well (Fisher's C=10.7, P=0.22, d.f.=8, n=375 plots). Data is based on a long, experimental species richness gradient ranging from monocultures to mixtures of 24 tree species. Examined variables are shown as boxes and relationships as directional arrows with significant positive effects in blue, significant negative effects in red and non-significant paths in dotted grey. Standardized (significant) path-coefficients are shown next to each path with asterisks indicating significance (* P<0.05, ** P<0.01, *** P<0.001), path-width is scaled by coefficient size. Significant partial correlations are shown through grey, bi-directional arrows. Species richness was $\log_2$ transformed while asynchrony, μ_{AWP} and σ_{AWP} were square-root transformed. The variation in asynchrony, μ_{AWP} and σ_{AWP} explained by fixed (left, marginal R^2) and fixed together with random model effects (right, conditional R^2) is shown in the corresponding boxes.

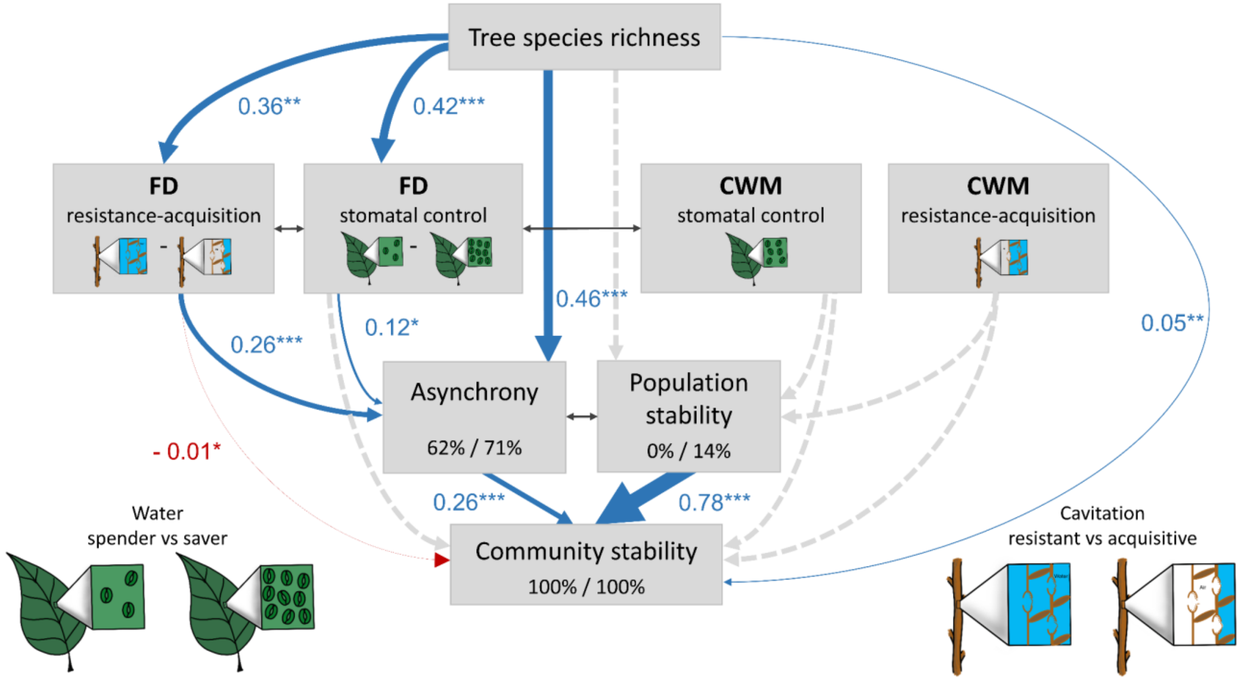

Fig. S16. Direct and indirect effects of tree species richness, drought-tolerance diversity and CWMs of drought-tolerance traits on community stability. The structural equation model (SEM) tests the direct effects of tree species richness as well as its indirect effects mediated via asynchrony and population stability on community stability (H1) and is fit without square-root transformed asynchrony, population stability and community stability but with an exponential variance structure (varExp) (*67*) for $\log_2$ species richness. Effects of functional diversity are explored through testing the effect of functional diversity of stomatal control (FD stomatal control) and functional diversity of resistance-acquisition (FD resistance-acquisition) as well as their indirect effects mediated via asynchrony on community stability (H2). Effects of CWM traits are explored through testing the effect of the CWM of stomatal control (CWM stomatal control) and the CWM of resistance-acquisition (CWM resistance-acquisition) as well as their indirect effects mediated via population stability on community stability (H3). The sketches schematically illustrate the trait gradients: water-spending vs water-saving stomatal control (few vs abundant stomata) and resistant vs acquisitive (high versus low cavitation resistance). Functional diversity was calculated as abundance-weighted functional dispersion. The SEM fit to the data was only marginally significant (Fisher's C=9.2, P=0.51, d.f.=10, n=218 plots). Data is based on a long, experimental species richness gradient with mixtures of 2, 4, 8, 16 and 24 tree species. Examined variables are shown as boxes and relationships as directional arrows with significant positive effects in blue, significant negative effects in red and non-significant paths in dotted grey based on a hypothesis driven SEM framework (Fig. S8). Standardized (significant) path-coefficients are shown next to each path with asterisks indicating significance (* P<0.05, ** P<0.01, *** P<0.001), path-width is scaled by coefficient size. Significant partial correlations are shown through grey, bi-directional arrows. Species richness was $\log_2$ transformed. The variation in asynchrony and community stability explained by fixed (left, marginal R^2) and fixed together with random model effects (right, conditional R^2) is shown in the corresponding boxes.

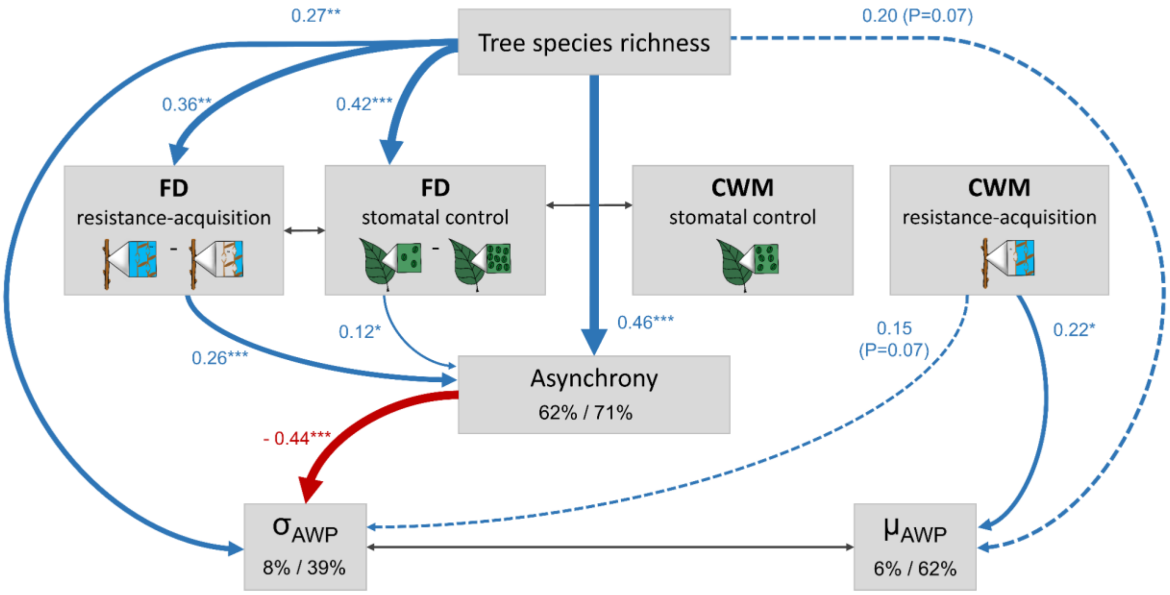

Fig. S17. Partitioning of the direct and indirect effects of species richness, drought-tolerance diversity and CWMs of drought-tolerance traits into overyielding and variance buffering effects of diversity. The structural equation model (SEM) separates the hypothesized effects of diversity on community stability (Fig. S8) into effects on the temporal mean (μ_{AWP}) and the temporal standard deviation of productivity (σ_{AWP}) and is fit without square-root transformed asynchrony, μ_{AWP} and σ_{AWP} but with an exponential variance structure (varExp) (*67*) for $\log_2$ species richness. Increases in μ_{AWP} enhance community stability through overyielding – a higher productivity in mixtures vs monocultures - and decreases in σ_{AWP} enhance community stability through buffered variations in productivity. All drivers hypothesized to influence community stability — species richness, functional diversity of stomatal control (FD stomatal control), functional diversity of resistance-acquisition (FD resistance-acquisition), the CWM of stomatal control (CWM stomatal control), the CWM of resistance-acquisition (CWM resistance-acquisition) and asynchrony — were tested for their effects on μ_{AWP} and σ_{AWP}. Only significant pathways ($P<0.05$) are shown here to avoid overplotting (see Fig. S11 for the full model). Population stability was not included in this analysis, as it did not respond to diversity nor CWM traits (Fig. 4). The sketches schematically illustrate the trait gradients: water-spending vs water-saving stomatal control (few versus abundant stomata) and resistant vs acquisitive (high versus low cavitation resistance). The SEM fit to the data was only marginally significant (Fisher's C=14.1, P=0.079, d.f.=8, n=218 plots). Data is based on a long, experimental species richness gradient with mixtures of 2, 4, 8, 16 and 24 tree species. Examined variables are shown as boxes and relationships as directional arrows with significant positive effects in blue, significant negative effects in red and non-significant paths in dotted grey. Standardized (significant) path-coefficients are shown next to each path with asterisks indicating significance (* $P<0.05$, ** $P<0.01$, *** $P<0.001$), path-width is scaled by coefficient size. Significant partial correlations are shown through grey, bi-directional arrows. Species richness was $\log_2$ transformed. The variation in asynchrony, μ_{AWP} and σ_{AWP} explained by fixed (left, marginal R^2) and fixed together with random model effects (right, conditional R^2) is shown in the corresponding boxes.

Table S1. Drought resistance and stomatal-control traits as well as traits of the leaf economics spectrum (LES) (*35*) used in this study.

Acronym	Trait description	Unit
Ψ_{50}	Water potential at which 50% initial conductivity is lost	MPa
VPDMAXFIT	Water pressure deficit (VPD) at CONMAXFIT	hPA
VPDPOI	VPD at the point of inflection of modelled stomatal conductance	hPA
CONMAXFIT	Modelled maximum stomata conductance	Non-dimensional
STOMDENS	Stomata density	1 mm^{-2}
STOIND	Product of STODENS and stomata size in μm^2	ratio
SLA	Specific leaf area	$m^2\ kg^{-1}$
LEAFT	Leaf toughness	N mm^{-1}
CN	Carbon to nitrogen ratio	Ratio

Note: All traits were measured on site and used to calculate species level mean trait values by refs. (*33*, *34*). See these studies for a detailed explication of the selected traits and Fig. S1 for additional information. In brief, we quantified stomatal control as species-specific stomatal sensitivity to water shortage via modelled curves of stomatal conductance (g_s) under increasing water pressure deficits (VPD) (*34*). In these curves, VPDMAXFIT is the VPD at maximum stomatal conductance of a species and represents the threshold at which a species starts to limit stomatal conductance due to increasing VPD (*34*). VPDPOI is the VPD at the second point of inflection of this modelled curve of stomatal conductance, that is the point when the slope of the g_s and VPD curve turns from negative to positive, and thus, can be taken as a measure of how quickly stomata close at high VPD, or in other words, of stomatal sensitivity. We consider both as physiological traits indicative of species-specific stomatal-control strategies: water spenders downregulate stomatal conductance only at high VPD while water savers close their stomata and down regulate stomatal conductance fast during increasing water shortage (Fig. S1). We have also included g_{smax} in the analysis (called CONMAXFIT, which is the modelled maximum conductance, see Kröber & Bruelheide (*34*)). However, in the BEF-China experiment, tree species' CONMAXFIT was uncorrelated to stomata size and stomata density but positively related to leaf area (i.e. to the leaf economics spectrum, see Fig. 4a in Kröber & Bruelheide (*34*)). For that reason, CONMAXFIT (as well as observed g_{smax} values) does not align well with our two PCA axes in Fig. S1. In contrast, VPDMAXFIT, which is the corresponding x value of the modelled g_s curve, perfectly aligns with PC1, which is the water spender - water saver axis. Morphological stomata traits are stomata density and stomata index, the product of stomata size and stomata density.

Table S2. Mixed-effects models exploring bivariate relationships between community stability, asynchrony, population stability and different facets of drought-tolerance diversity and CWMs of drought-tolerance traits.

Response	Fixed Effects	ddf	t	P-value	n	R^2_m	R^2_c
**All plots**							
Community stability	**Species richness**	132	3.98	**0.000**	375	0.06	0.26
**Mixtures only**							
Community stability	**Species richness**	87	3.72	**0.000**	218	0.08	0.22
Community stability	**Asynchrony**	95	10.13	**0.000**	218	0.34	0.54
Community stability	**Population stability**	95	26.30	**0.000**	218	0.77	0.82
Community stability	**FD stomatal control**	95	1.92	**0.058**	218	0.02	0.22
Community stability	FD resistance-acquisition	95	1.12	0.270	218	0.01	0.21
Community stability	CWM stomatal control	95	-0.45	0.652	218	0.00	0.21
Community stability	CWM resistance-acquisition	95	-0.15	0.880	218	0.00	0.21
Asynchrony	**Species richness**	87	9.53	**0.000**	218	0.38	0.49
Asynchrony	**FD stomatal control**	95	5.29	**0.000**	218	0.16	0.49
Asynchrony	**FD resistance-acquisition**	95	5.84	**0.000**	218	0.17	0.43
Population stability	Species richness	87	0.27	0.785	218	0.00	0.31
Population stability	CWM stomatal control	95	-0.65	0.515	218	0.00	0.31
Population stability	CWM resistance-acquisition	95	-0.36	0.719	218	0.00	0.31

Note: Significant fixed effects ($P<0.05$) printed in bold. Drought-tolerance diversity was quantified as functional diversity of stomatal control (FD stomatal control) and functional diversity of resistance-acquisition strategies (FD resistance-acquisition) and CWMs of drought-tolerance traits as CWM of stomatal control (CWM stomatal control) and of resistance-acquisition traits (CWM resistance-acquisition). Data is based on a planted diversity gradient ranging from monocultures up to mixtures of 24 tree species. Tree species richness was $\log_2$ transformed in all models. ddf are the denominator degrees of freedom; t the ratio between the estimate and its standard error; P-value from a t-distribution; n the number of plots; marginal R^2 values (R^2_m) show the variance explained by fixed effects and conditional (R^2_c) values the variance explained by fixed and random effects (*71*).

Table S3. List of the 40 planted broadleaved evergreen and deciduous tree species.

Species names	Family	Species code	Leaf habit	Site
Acer davidii	*Sapindaceae*	27	D	A
Ailanthus altissima	*Simaroubaceae*	29	D	B
Alniphyllum fortunei	*Styracaceae*	30	D	B
Betula luminifera	*Betulaceae*	31	D	B
Castanea henryi	*Fagaceae*	1	D	A
Castanopsis carlesii	*Fagaceae*	10	E	A
Castanopsis eyrei	*Fagaceae*	13	E	AB
Castanopsis fargesii	*Fagaceae*	32	E	B
Castanopsis sclerophylla	*Fagaceae*	14	E	AB
Celtis biondii	*Cannabaceae*	33	D	B
Choerospondias axillaris	*Anacardiaceae*	4	D	A
Cinnamomum camphora	*Lauraceae*	17	E	AB
Cyclobalanopsis glauca	*Fagaceae*	11	E	AB
Cyclobalanopsis myrsinifolia	*Fagaceae*	9	E	A
Daphniphyllum oldhamii	*Daphniphyllaceae*	16	E	AB
Diospyros japonica	*Ebenaceae*	15	D	AB
Elaeocarpus chinensis	*Elaeocarpaceae*	34	E	B
Elaeocarpus glabripetalus	*Elaeocarpaceae*	35	E	B
Elaeocarpus japonicus	*Elaeocarpaceae*	36	E	B
Idesia polycarpa	*Salicaceae*	37	D	B
Koelreuteria bipinnata	*Sapindaceae*	18	D	A
Liquidambar formosana	*Altingiaceae*	6	D	A
Lithocarpus glaber	*Fagaceae*	12	E	AB
Machilus grijsii	*Lauraceae*	39	E	B
Machilus leptophylla	*Lauraceae*	41	E	B
Machilus thunbergii	*Lauraceae*	40	E	B
Manglietia fordiana	*Magnoliaceae*	42	E	B
Melia azedarach	*Meliaceae*	26	D	A
Meliosma flexuosa	*Sabiaceae*	38	D	B
Nyssa sinensis	*Cornaceae*	20	D	A
Phoebe bournei	*Lauraceae*	43	E	B
Quercus acutissima	*Fagaceae*	25	D	A
Quercus fabri	*Fagaceae*	24	D	A
Quercus phillyreoides	*Fagaceae*	44	E	B
Quercus serrata	*Fagaceae*	8	D	A
Rhus chinensis	*Anacardiaceae*	23	D	A
Sapindus saponaria	*Sapindaceae*	19	D	A
Triadica cochinchinensis	*Euphorbiaceae*	22	D	A
Triadica sebifera	*Euphorbiaceae*	21	D	A
Schima superba	*Theaceae*	3	E	AB

Note: Shown are species and family names, species identity codes, leaf habit (E, evergreen; D, Deciduous) and the site at which the species were planted. For more details on the characteristics and taxonomy of the tree species see ref. (*16*) and for the experimental design refs. (16, 39).

REFERENCES AND NOTES

1. IPCC, *Climate Change 2014, Impacts, Adaptation, and Vulnerability. Part A: Global and Sectoral Aspects. Contribution of Working Group II to the Fifth Assessment Report of the Intergovernmental Panel on Climate Change* (Cambridge Univ. Press, 2014).

2. B. Choat, S. Jansen, T. J. Brodribb, H. Cochard, S. Delzon, R. Bhaskar, S. J. Bucci, T. S. Feild, S. M. Gleason, U. G. Hacke, A. L. Jacobsen, F. Lens, H. Maherali, J. Martínez-Vilalta, S. Mayr, M. Mencuccini, P. J. Mitchell, A. Nardini, J. Pittermann, R. B. Pratt, J. S. Sperry, M. Westoby, I. J. Wright, A. E. Zanne, Global convergence in the vulnerability of forests to drought. *Nature* **491**, 752–755 (2012).

3. W. R. L. Anderegg, A. T. Trugman, G. Badgley, C. M. Anderson, A. Bartuska, P. Ciais, D. Cullenward, C. B. Field, J. Freeman, S. J. Goetz, J. A. Hicke, D. Huntzinger, R. B. Jackson, J. Nickerson, S. Pacala, J. T. Randerson, Climate-driven risks to the climate mitigation potential of forests. *Science* **368** eaaz7005 (2020).

4. B. W. Griscom, J. Adams, P. W. Ellis, R. A. Houghton, G. Lomax, D. A. Miteva, W. H. Schlesinger, D. Shoch, J. V. Siikamäki, P. Smith, P. Woodbury, C. Zganjar, A. Blackman, J. Campari, R. T. Conant, C. Delgado, P. Elias, T. Gopalakrishna, M. R. Hamsik, M. Herrero, J. Kiesecker, E. Landis, L. Laestadius, S. M. Leavitt, S. Minnemeyer, S. Polasky, P. Potapov, F. E. Putz, J. Sanderman, M. Silvius, E. Wollenberg, J. Fargione, Natural climate solutions. *Proc. Natl. Acad. Sci. U.S.A.* **114**, 11645–11650 (2017).

5. T. Jucker, O. Bouriaud, D. Avacaritei, D. A. Coomes, Stabilizing effects of diversity on aboveground wood production in forest ecosystems: Linking patterns and processes. *Ecol. Lett.* **17**, 1560–1569 (2014).

6. C. Messier, J. Bauhus, R. Sousa-Silva, H. Auge, L. Baeten, N. Barsoum, H. Bruelheide, B. Caldwell, J. Cavender-Bares, E. Dhiedt, N. Eisenhauer, G. Ganade, D. Gravel, J. Guillemot, J. S. Hall, A. Hector, B. Hérault, H. Jactel, J. Koricheva, H. Kreft, S. Mereu, B. Muys, C. A. Nock, A. Paquette, J. D. Parker, M. P. Perring, Q. Ponette, C. Potvin, P. B. Reich, M. Scherer-Lorenzen, F. Schnabel, K. Verheyen, M. Weih, M. Wollni, D. C. Zemp, For the sake of resilience and multifunctionality, let's diversify planted forests! *Conserv. Lett.* e12829 (2021).

7. D. Craven, N. Eisenhauer, W. D. Pearse, Y. Hautier, F. Isbell, C. Roscher, M. Bahn, C. Beierkuhnlein, G. Bönisch, N. Buchmann, C. Byun, J. A. Catford, B. E. L. Cerabolini, J. H. C. Cornelissen, J. M. Craine, E. de Luca, A. Ebeling, J. N. Griffin, A. Hector, J. Hines, A. Jentsch, J. Kattge, J. Kreyling, V. Lanta, N. Lemoine, S. T. Meyer, V. Minden, V. Onipchenko, H. W. Polley, P. B. Reich, J. van Ruijven, B. Schamp, M. D. Smith, N. A. Soudzilovskaia, D. Tilman, A. Weigelt, B. Wilsey, P. Manning, Multiple facets of biodiversity drive the diversity–stability relationship. *Nat. Ecol. Evol.* **2**, 1579–1587 (2018).

8. F. Isbell, D. Craven, J. Connolly, M. Loreau, B. Schmid, C. Beierkuhnlein, T. M. Bezemer, C. Bonin, H. Bruelheide, E. de Luca, A. Ebeling, J. N. Griffin, Q. Guo, Y. Hautier, A. Hector, A. Jentsch, J. Kreyling, V. Lanta, P. Manning, S. T. Meyer, A. S. Mori, S. Naeem, P. A.

Niklaus, H. W. Polley, P. B. Reich, C. Roscher, E. W. Seabloom, M. D. Smith, M. P. Thakur, D. Tilman, B. F. Tracy, van der Putten, Wim H., J. van Ruijven, A. Weigelt, W. W. Weisser, B. Wilsey, N. Eisenhauer, Biodiversity increases the resistance of ecosystem productivity to climate extremes. *Nature* **526**, 574–577 (2015).

9. D. Tilman, P. B. Reich, J. M. H. Knops, Biodiversity and ecosystem stability in a decade-long grassland experiment. *Nature* **441**, 629–632 (2006).

10. A. Hector, Y. Hautier, P. Saner, L. Wacker, R. Bagchi, J. Joshi, M. Scherer-Lorenzen, E. M. Spehn, E. Bazeley-White, M. Weilenmann, M. C. Caldeira, P. G. Dimitrakopoulos, J. A. Finn, K. Huss-Danell, A. Jumpponen, C. P. H. Mulder, C. Palmborg, J. S. Pereira, A. S. D. Siamantziouras, A. C. Terry, A. Y. Troumbis, B. Schmid, M. Loreau, General stabilizing effects of plant diversity on grassland productivity through population asynchrony and overyielding. *Ecology* **91**, 2213–2220 (2010).

11. M. del Río, H. Pretzsch, R. Ruíz-Peinado, E. Ampoorter, P. Annighöfer, I. Barbeito, K. Bielak, G. Brazaitis, L. Coll, L. Drössler, M. Fabrika, D. I. Forrester, M. Heym, V. Hurt, V. Kurylyak, M. Löf, F. Lombardi, E. Madrickiene, B. Matović, F. Mohren, R. Motta, J. Ouden, M. Pach, Q. Ponette, G. Schütze, J. Skrzyszewski, V. Sramek, H. Sterba, D. Stojanović, M. Svoboda, T. M. Zlatanov, A. Bravo-Oviedo, Species interactions increase the temporal stability of community productivity in *Pinus sylvestris–Fagus sylvatica* mixtures across Europe. *J. Ecol.* **105**, 1032–1043 (2017).

12. X. Morin, L. Fahse, C. de Mazancourt, M. Scherer-Lorenzen, H. Bugmann, Temporal stability in forest productivity increases with tree diversity due to asynchrony in species dynamics. *Ecol. Lett.* **17**, 1526–1535 (2014).

13. F. Schnabel, J. A. Schwarz, A. Dănescu, A. Fichtner, C. A. Nock, J. Bauhus, C. Potvin, Drivers of productivity and its temporal stability in a tropical tree diversity experiment. *Glob. Chang. Biol.* **25**, 4257–4272 (2019).

14. S. Yachi, M. Loreau, Biodiversity and ecosystem productivity in a fluctuating environment: The insurance hypothesis. *Proc. Natl. Acad. Sci. U.S.A.* **96**, 1463–1468 (1999).

15. D. Tilman, The ecological consequences of changes in biodiversity: A search for general principles. *Ecology* **80**, 1455–1474 (1999).

16. Y. Huang, Y. Chen, N. Castro-Izaguirre, M. Baruffol, M. Brezzi, A. Lang, Y. Li, W. Härdtle, G. von Oheimb, X. Yang, X. Liu, K. Pei, S. Both, B. Yang, D. Eichenberg, T. Assmann, J. Bauhus, T. Behrens, F. Buscot, X.-Y. Chen, D. Chesters, B.-Y. Ding, W. Durka, A. Erfmeier, J. Fang, M. Fischer, L.-D. Guo, D. Guo, J. L. M. Gutknecht, J.-S. He, C.-L. He, A. Hector, L. Hönig, R.-Y. Hu, A.-M. Klein, P. Kühn, Y. Liang, S. Li, S. Michalski, M. Scherer-Lorenzen, K. Schmidt, T. Scholten, A. Schuldt, X. Shi, M.-Z. Tan, Z. Tang, S. Trogisch, Z. Wang, E. Welk, C. Wirth, T. Wubet, W. Xiang, M. Yu, X.-D. Yu, J. Zhang, S. Zhang, N. Zhang, H.-Z. Zhou, C.-D. Zhu, L. Zhu, H. Bruelheide, K. Ma, P. A. Niklaus, B. Schmid, Impacts of species richness on productivity in a large-scale subtropical forest experiment. *Science* **362**, 80–83 (2018).

17. F. van der Plas, Biodiversity and ecosystem functioning in naturally assembled communities. *Biol. Rev.* **94**, 1220–1245 (2019).

18. M. Loreau, C. de Mazancourt, Species synchrony and its drivers: Neutral and nonneutral community dynamics in fluctuating environments. *Am. Nat.* **172**, E48-E66 (2008).

19. M. Loreau, C. de Mazancourt, Biodiversity and ecosystem stability: A synthesis of underlying mechanisms. *Ecol. Lett.* **16**, 106–115 (2013).

20. E. Valencia, F. de Bello, T. Galland, P. B. Adler, J. Lepš, A. E-Vojtkó, R. van Klink, C. P. Carmona, J. Danihelka, J. Dengler, D. J. Eldridge, M. Estiarte, R. García-González, E. Garnier, D. Gómez-García, S. P. Harrison, T. Herben, R. Ibáñez, A. Jentsch, N. Juergens, M. Kertész, K. Klumpp, F. Louault, R. H. Marrs, R. Ogaya, G. Ónodi, R. J. Pakeman, I. Pardo, M. Pärtel, B. Peco, J. Peñuelas, R. F. Pywell, M. Rueda, W. Schmidt, U. Schmiedel, M. Schuetz, H. Skálová, P. Šmilauer, M. Šmilauerová, C. Smit, M. Song, M. Stock, J. Val, V. Vandvik, D. Ward, K. Wesche, S. K. Wiser, B. A. Woodcock, T. P. Young, F.-H. Yu, M. Zobel, L. Götzenberger, Synchrony matters more than species richness in plant community stability at a global scale. *Proc. Natl. Acad. Sci. U.S.A.* **117**, 24345–24351 (2020).

21. Z. Yuan, A. Ali, S. Wang, X. Wang, F. Lin, Y. Wang, S. Fang, Z. Hao, M. Loreau, L. Jiang, Temporal stability of aboveground biomass is governed by species asynchrony in temperate forests. *Ecol. Indic.* **107**, 105661 (2019).

22. M. Mund, W. L. Kutsch, N. Winiger, T. Kahl, A. Knohl, M. V. Skomarkova, E.-D. Schulze, The influence of climate and fructification on the inter-annual variability of stem growth and net primary productivity in an old-growth, mixed beech forest. *Tree Physiol.* **30**, 689–704 (2010).

23. Y. Du, B. Yang, S.-C. Chen, K. Ma, Diverging shifts in spring phenology in response to biodiversity loss in a subtropical forest. *J. Veg. Sci.* **30**, 1175–1183 (2019).

24. N. McDowell, W. T. Pockman, C. D. Allen, D. D. Breshears, N. Cobb, T. Kolb, J. Plaut, J. Sperry, A. West, D. G. Williams, E. A. Yepez, Mechanisms of plant survival and mortality during drought: Why do some plants survive while others succumb to drought? *New Phytol.* **178**, 719–739 (2008).

25. A. T. Tredennick, C. de Mazancourt, M. Loreau, P. B. Adler, Environmental responses, not species interactions, determine synchrony of dominant species in semiarid grasslands. *Ecology* **98**, 971–981 (2017).

26. K. E. Barry, L. Mommer, J. van Ruijven, C. Wirth, A. J. Wright, Y. Bai, J. Connolly, G. B. de Deyn, H. de Kroon, F. Isbell, A. Milcu, C. Roscher, M. Scherer-Lorenzen, B. Schmid, A. Weigelt, The future of complementarity: Disentangling causes from consequences. *Trends Ecol. Evol.* **34**, 167–180 (2019).

27. C. Grossiord, Having the right neighbors: How tree species diversity modulates drought impacts on forests. *New Phytol.* **228**, 42–49 (2020).

28. L. M. Thibaut, S. R. Connolly, Understanding diversity-stability relationships: Towards a unified model of portfolio effects. *Ecol. Lett.* **16**, 140–150 (2013).

29. Q. Xu, X. Yang, Y. Yan, S. Wang, M. Loreau, L. Jiang, Consistently positive effect of species diversity on ecosystem, but not population, temporal stability. *Ecol. Lett.* **24**, 2256–2266 (2021).

30. C. D. Allen, A. K. Macalady, H. Chenchouni, D. Bachelet, N. McDowell, M. Vennetier, T. Kitzberger, A. Rigling, D. D. Breshears, E. H. Hogg, P. Gonzalez, R. Fensham, Z. Zhang, J. Castro, N. Demidova, J.-H. Lim, G. Allard, S. W. Running, A. Semerci, N. Cobb, A global overview of drought and heat-induced tree mortality reveals emerging climate change risks for forests. *For. Ecol. Manage.* **259**, 660–684 (2010).

31. B. Schuldt, A. Buras, M. Arend, Y. Vitasse, C. Beierkuhnlein, A. Damm, M. Gharun, T. E. Grams, M. Hauck, P. Hajek, H. Hartmann, E. Hilbrunner, G. Hoch, M. Holloway-Phillips, C. Körner, E. Larysch, T. Lübbe, D. B. Nelson, A. Rammig, A. Rigling, L. Rose, N. K. Ruehr, K. Schumann, F. Weiser, C. Werner, T. Wohlgemuth, C. S. Zang, A. Kahmen, A first assessment of the impact of the extreme 2018 summer drought on Central European forests. *Basic Appl. Ecol.* **45**, 86–103 (2020).

32. J. Martínez-Vilalta, N. Garcia-Forner, Water potential regulation, stomatal behaviour and hydraulic transport under drought: Deconstructing the iso/anisohydric concept. *Plant Cell Environ.* **40**, 962–976 (2017).

33. W. Kröber, S. Zhang, M. Ehmig, H. Bruelheide, Linking xylem hydraulic conductivity and vulnerability to the leaf economics spectrum--a cross-species study of 39 evergreen and deciduous broadleaved subtropical tree species. *PLOS ONE* **9**, e109211 (2014).

34. W. Kröber, H. Bruelheide, Transpiration and stomatal control: A cross-species study of leaf traits in 39 evergreen and deciduous broadleaved subtropical tree species. *Trees* **28**, 901–914 (2014).

35. P. B. Reich, The world-wide 'fast-slow' plant economics spectrum: A traits manifesto. *J. Ecol.* **102**, 275–301 (2014).

36. A. Fichtner, F. Schnabel, H. Bruelheide, M. Kunz, K. Mausolf, A. Schuldt, W. Härdtle, G. von Oheimb, Neighbourhood diversity mitigates drought impacts on tree growth. *J. Ecol.* **108**, 865–875 (2020).

37. R. S. Oliveira, C. B. Eller, F. de V. Barros, M. Hirota, M. Brum, P. Bittencourt, Linking plant hydraulics and the fast–slow continuum to understand resilience to drought in tropical ecosystems. *New Phytol.* **230**, 904–923 (2021).

38. M. Májeková, F. de Bello, J. Doležal, J. Lepš, Plant functional traits as determinants of population stability. *Ecology* **95**, 2369–2374 (2014).

39. H. Bruelheide, K. Nadrowski, T. Assmann, J. Bauhus, S. Both, F. Buscot, X.-Y. Chen, B. Ding, W. Durka, A. Erfmeier, J. L. M. Gutknecht, D. Guo, L.-D. Guo, W. Härdtle, J.-S. He,

A.-M. Klein, P. Kühn, Y. Liang, X. Liu, S. Michalski, P. A. Niklaus, K. Pei, M. Scherer-Lorenzen, T. Scholten, A. Schuldt, G. Seidler, S. Trogisch, G. von Oheimb, E. Welk, C. Wirth, T. Wubet, X. Yang, M. Yu, S. Zhang, H.-Z. Zhou, M. Fischer, K. Ma, B. Schmid, Designing forest biodiversity experiments: General considerations illustrated by a new large experiment in subtropical China. *Methods Ecol. Evol.* **5**, 74–89 (2014).

40. J. S. Lefcheck, piecewiseSEM: Piecewise structural equation modelling in r for ecology, evolution, and systematics. *Methods Ecol. Evol.* **7**, 573–579 (2016).

41. F. J. Bongers, B. Schmid, H. Bruelheide, F. Bongers, S. Li, G. von Oheimb, Y. Li, A. Cheng, K. Ma, X. Liu, Functional diversity effects on productivity increase with age in a forest biodiversity experiment. *Nat. Ecol. Evol.* 10.1038/s41559-021-01564-3 (2021).

42. S. Trogisch, X. Liu, G. Rutten, K. Xue, J. Bauhus, U. Brose, W. Bu, S. Cesarz, D. Chesters, J. Connolly, X. Cui, N. Eisenhauer, L. Guo, S. Haider, W. Härdtle, M. Kunz, L. Liu, Z. Ma, S. Neumann, W. Sang, A. Schuldt, Z. Tang, N. M. van Dam, G. von Oheimb, M.-Q. Wang, S. Wang, A. Weinhold, C. Wirth, T. Wubet, X. Xu, B. Yang, N. Zhang, C.-D. Zhu, K. Ma, Y. Wang, H. Bruelheide, The significance of tree-tree interactions for forest ecosystem functioning. *Basic Appl. Ecol.* **55**, 33–52 (2021).

43. A. Fichtner, W. Härdtle, Y. Li, H. Bruelheide, M. Kunz, G. von Oheimb, From competition to facilitation: How tree species respond to neighbourhood diversity. *Ecol. Lett.* **20**, 892–900 (2017).

44. M. J. O'Brien, G. Reynolds, R. Ong, A. Hector, Resistance of tropical seedlings to drought is mediated by neighbourhood diversity. *Nat. Ecol. Evol.* **1**, 1643–1648 (2017).

45. Y. Yan, J. Connolly, M. Liang, L. Jiang, S. Wang, Mechanistic links between biodiversity effects on ecosystem functioning and stability in a multi-site grassland experiment. *J. Ecol.* **109**, 3370–3378 (2021).

46. D. I. Forrester, J. Bauhus, A review of processes behind diversity—Productivity relationships in forests. *Curr. Forestry Rep.* **2**, 45–61 (2016).

47. J. Guillemot, M. Kunz, F. Schnabel, A. Fichtner, C. P. Madsen, T. Gebauer, W. Härdtle, G. von Oheimb, C. Potvin, Neighbourhood-mediated shifts in tree biomass allocation drive overyielding in tropical species mixtures. *New Phytol.* **228**, 1256–1268 (2020).

48. W. R. L. Anderegg, A. G. Konings, A. T. Trugman, K. Yu, D. R. Bowling, R. Gabbitas, D. S. Karp, S. Pacala, J. S. Sperry, B. N. Sulman, N. Zenes, Hydraulic diversity of forests regulates ecosystem resilience during drought. *Nature* **561**, 538–541 (2018).

49. H. D. Adams, M. J. B. Zeppel, W. R. L. Anderegg, H. Hartmann, S. M. Landhäusser, D. T. Tissue, T. E. Huxman, P. J. Hudson, T. E. Franz, C. D. Allen, L. D. L. Anderegg, G. A. Barron-Gafford, D. J. Beerling, D. D. Breshears, T. J. Brodribb, H. Bugmann, R. C. Cobb, A. D. Collins, L. T. Dickman, H. Duan, B. E. Ewers, L. Galiano, D. A. Galvez, N. Garcia-Forner, M. L. Gaylord, M. J. Germino, A. Gessler, U. G. Hacke, R. Hakamada, A. Hector, M. W. Jenkins, J. M. Kane, T. E. Kolb, D. J. Law, J. D. Lewis, J.-M. Limousin, D. M. Love,

A. K. Macalady, J. Martínez-Vilalta, M. Mencuccini, P. J. Mitchell, J. D. Muss, M. J. O'Brien, A. P. O'Grady, R. E. Pangle, E. A. Pinkard, F. I. Piper, J. A. Plaut, W. T. Pockman, J. Quirk, K. Reinhardt, F. Ripullone, M. G. Ryan, A. Sala, S. Sevanto, J. S. Sperry, R. Vargas, M. Vennetier, D. A. Way, C. Xu, E. A. Yepez, N. G. McDowell, A multi-species synthesis of physiological mechanisms in drought-induced tree mortality. *Nat. Ecol. Evol.* **1**, 1285–1291 (2017).

50. D. I. Forrester, in *Mixed-Species Forests, Ecology and Management*, H. Pretzsch, D. I. Forrester, J. Bauhus, Eds. (Springer Nature, 2017), pp. 73–115.

51. C. Scoffoni, D. S. Chatelet, J. Pasquet-Kok, M. Rawls, M. J. Donoghue, E. J. Edwards, L. Sack, Hydraulic basis for the evolution of photosynthetic productivity. *Nat. Plants* **2**, 16072 (2016).

52. S. Hoeber, C. Leuschner, L. Köhler, D. Arias-Aguilar, B. Schuldt, The importance of hydraulic conductivity and wood density to growth performance in eight tree species from a tropical semi-dry climate. *For. Ecol. Manage.* **330**, 126–136 (2014).

53. M. J. O’Brien, S. Leuzinger, C. D. Philipson, J. Tay, A. Hector, Drought survival of tropical tree seedlings enhanced by non-structural carbohydrate levels. *Nat. Clim. Chang.* **4**, 710–714 (2014).

54. M. Kunz, A. Fichtner, W. Härdtle, P. Raumonen, H. Bruelheide, G. von Oheimb, Neighbour species richness and local structural variability modulate aboveground allocation patterns and crown morphology of individual trees. *Ecol. Lett.* **22**, 2130–2140 (2019).

55. W. Bu, B. Schmid, X. Liu, Y. Li, W. Härdtle, G. von Oheimb, Y. Liang, Z. Sun, Y. Huang, H. Bruelheide, K. Ma, Interspecific and intraspecific variation in specific root length drives aboveground biodiversity effects in young experimental forest stands. *J. Plant Ecol.* **10**, 158–169 (2017).

56. L. Schwendenmann, E. Pendall, R. Sanchez-Bragado, N. Kunert, D. Hölscher, Tree water uptake in a tropical plantation varying in tree diversity: Interspecific differences, seasonal shifts and complementarity. *Ecohydrology* **8**, 1–12 (2015).

57. J. R. Lasky, M. Uriarte, V. K. Boukili, D. L. Erickson, W. John Kress, R. L. Chazdon, The relationship between tree biodiversity and biomass dynamics changes with tropical forest succession. *Ecol. Lett.* **17**, 1158–1167 (2014).

58. X. Yang, J. Bauhus, S. Both, T. Fang, W. Härdtle, W. Kröber, K. Ma, K. Nadrowski, K. Pei, M. Scherer-Lorenzen, T. Scholten, G. Seidler, B. Schmid, G. von Oheimb, H. Bruelheide, Establishment success in a forest biodiversity and ecosystem functioning experiment in subtropical China (BEF-China). *Eur. J. Forest. Res.* **132**, 593–606 (2013).

59. X.-H. Wang, M. Kent, X.-F. Fang, Evergreen broad-leaved forest in Eastern China: Its ecology and conservation and the importance of resprouting in forest restoration. *For. Ecol. Manage.* **245**, 76–87 (2007).

60. M.-M. Shi, S. G. Michalski, E. Welk, X.-Y. Chen, W. Durka, Phylogeography of a widespread Asian subtropical tree: Genetic east-west differentiation and climate envelope modelling suggest multiple glacial refugia. *J. Biogeogr.* **41**, 1710–1720 (2014).

61. A. Fichtner, W. Härdtle, H. Bruelheide, M. Kunz, Y. Li, G. von Oheimb, Neighbourhood interactions drive overyielding in mixed-species tree communities. *Nat. Commun.* **9**, 1144 (2018).

62. Y. Hautier, E. W. Seabloom, E. T. Borer, P. B. Adler, W. S. Harpole, H. Hillebrand, E. M. Lind, A. S. MacDougall, C. J. Stevens, J. D. Bakker, Y. M. Buckley, C. Chu, S. L. Collins, P. Daleo, E. I. Damschen, K. F. Davies, P. A. Fay, J. Firn, D. S. Gruner, V. L. Jin, J. A. Klein, J. M. H. Knops, K. J. La Pierre, W. Li, R. L. McCulley, B. A. Melbourne, J. L. Moore, L. R. O'Halloran, S. M. Prober, A. C. Risch, M. Sankaran, M. Schuetz, A. Hector, Eutrophication weakens stabilizing effects of diversity in natural grasslands. *Nature* **508**, 521–525 (2014).

63. S. van Buuren, K. Groothuis-Oudshoorn, mice: Multivariate imputation by chained equations in R. *J. Stat. Softw.* **45**, 1–67 (2011).

64. J. Oksanen, F. G. Blanchet, M. Friendly, R. Kindt, P. Legendre, D. McGlinn, P. R. Minchin, R. B. O'Hara, G. L. Simpson, P. Solymos, M. Stevens, E. Szoecs, H. Wagner, *vegan: Community Ecology Package* (2019).

65. E. Laliberté, P. Legendre, A distance-based framework for measuring functional diversity from multiple traits. *Ecology* **91**, 299–305 (2010).

66. E. Laliberté, P. Legendre, B. Shipley, FD: Measuring functional diversity (FD) from multiple traits, and other tools for functional ecology, R package version 1.0-12. (2014).

67. J. Pinheiro, D. Bates, S. DebRoy, D. Sarkar, R Core Team, *nlme, Linear and Nonlinear Mixed Effects Models* (2020).

68. D. Lüdecke, ggeffects: Tidy data frames of marginal effects from regression models. *J. Open Source Softw.* **3**, 772 (2018).

69. C. F. Dormann, J. Elith, S. Bacher, C. Buchmann, G. Carl, G. Carré, J. R. G. Marquéz, B. Gruber, B. Lafourcade, P. J. Leitão, T. Münkemüller, C. McClean, P. E. Osborne, B. Reineking, B. Schröder, A. K. Skidmore, D. Zurell, S. Lautenbach, Collinearity: A review of methods to deal with it and a simulation study evaluating their performance. *Ecography* **36**, 27–46 (2013).

70. R Core Team, *R: A Language and Environment for Statistical Computing* (R Foundation for Statistical Computing, 2019).

71. S. Nakagawa, H. Schielzeth, R. B. O'Hara, A general and simple method for obtaining R^2 from generalized linear mixed-effects models. *Methods Ecol. Evol.* **4**, 133–142 (2013).

72. S. M. Vicente-Serrano, S. Beguería, J. I. López-Moreno, A multiscalar drought index sensitive to global warming: The standardized precipitation evapotranspiration index. *J. Climate* **23**, 1696–1718 (2010).

73. I. Harris, T. J. Osborn, P. Jones, D. Lister, Version 4 of the CRU TS monthly high-resolution gridded multivariate climate dataset. *Sci. Data* **7**, 109 (2020).

74. S. Beguería, S. M. Vicente-Serrano, *SPEI: Calculation of the Standardised Precipitation-Evapotranspiration Index* (2017).
